# Molecular neurobiology of loss: a role for basolateral amygdala extracellular matrix

**DOI:** 10.1038/s41380-023-02231-8

**Published:** 2023-08-29

**Authors:** Marissa A. Smail, Brittany L. Smith, Rammohan Shukla, Khaled Alganem, Hunter M. Eby, Justin L. Bollinger, Ria K. Parikh, James B. Chambers, James K. Reigle, Rachel D. Moloney, Nawshaba Nawreen, Eric S. Wohleb, Harry Pantazopoulos, Robert E. McCullumsmith, James P. Herman

**Affiliations:** 1 Department of Pharmacology and Systems Physiology, University of Cincinnati, Cincinnati, OH USA; 2 Neuroscience Graduate Program, University of Cincinnati, Cincinnati, OH USA; 3 Department of Neurosciences, University of Toledo, Toledo, OH USA; 4 Department of Biomedical Informatics, Cincinnati Children’s Hospital Medical Center, Cincinnati, OH USA; 5 School of Pharmacy, University College Cork, Cork, Ireland UK; 6 Department of Pharmacology and Therapeutics, University College Cork, Cork, Ireland UK; 7 APC Microbiome Ireland, University College Cork, Cork, Ireland UK; 8 Department of Neurobiology and Anatomical Sciences, University of Mississippi Medical Center, Jackson, MS USA; 9 Neurosciences Institute, ProMedica, Toledo, OH USA; 10 Veterans Affairs Medical Center, Cincinnati, OH USA; 11 Department of Neurology, University of Cincinnati, Cincinnati, OH USA

**Keywords:** Neuroscience, Molecular biology

## Abstract

Psychological loss is a common experience that erodes well-being and negatively impacts quality of life. The molecular underpinnings of loss are poorly understood. Here, we investigate the mechanisms of loss using an environmental enrichment removal (ER) paradigm in male rats. The basolateral amygdala (BLA) was identified as a region of interest, demonstrating differential Fos responsivity to ER and having an established role in stress processing and adaptation. A comprehensive multi-omics investigation of the BLA, spanning multiple cohorts, platforms, and analyses, revealed alterations in microglia and the extracellular matrix (ECM). Follow-up studies indicated that ER decreased microglia size, complexity, and phagocytosis, suggesting reduced immune surveillance. Loss also substantially increased ECM coverage, specifically targeting perineuronal nets surrounding parvalbumin interneurons, suggesting decreased plasticity and increased inhibition within the BLA following loss. Behavioral analyses suggest that these molecular effects are linked to impaired BLA salience evaluation, leading to a mismatch between stimulus and reaction intensity. These loss-like behaviors could be rescued by depleting BLA ECM during the removal period, helping us understand the mechanisms underlying loss and revealing novel molecular targets to ameliorate its impact.

## Introduction

Psychological loss is something that most people experience in their lifetime. Losing something of value (e.g., interpersonal relationships, financial stability, secure housing, health) can erode well-being and negatively impact quality of life [1–3]. The impact of loss is felt in particular during times of natural disasters, political upheavals, and pandemics [4].

Loss can be defined as a “state of deprivation of a motivationally significant conspecific, object, or situation [1].” This relative deprivation often precipitates a depressed emotional state, and symptoms of loss resemble those of major depressive disorder (MDD), including amotivation, sadness, withdrawal, and rumination [1–3]. Loss is also associated with “atypical” MDD symptoms such as weight gain and hypoactive hypothalamic–pituitary–adrenal (HPA) axis responsivity [5, 6]. Little is known about the molecular mechanisms underlying loss, as the unique phenotype is difficult to track clinically and receives little attention in preclinical studies [5–7]. Elucidating the molecular mechanisms driving the response to loss could reveal novel treatments that may be of widespread benefit.

Our lab recently developed a model of loss in rats, in which environmental enrichment (EE) serves as proxy for positive life events and its removal emulates loss [8, 9]. EE is known to have rewarding properties in rodents, providing social, physical, and cognitive stimulation that contribute to enhanced cognition and neuroplasticity, as well as reducing emotional reactivity to stress [10, 11]. The enrichment removal (ER) protocol begins with 4 weeks of EE followed by removal to single housing lacking positive stimuli. This negative transition from high to low environmental stimulation is consistent with removal of a motivationally significant condition. Indeed, after 1 week of ER, adult male rats exhibit “loss-like” phenotypes, including increased passive coping in the forced swim test, increased hedonic drive in the sucrose preference test, weight gain, and hypoactive HPA axis responsivity relative to EE and standard housed (SH) rats [8]. Thus, ER affords a unique opportunity to study loss-related pathologies in a rodent model.

Here, we utilize a series of complementary approaches to investigate the biological substrates of psychological loss. Post-behavior (forced swim) Fos expression revealed selective recruitment of the basolateral amygdala (BLA), a region with established roles in stress regulation and behavioral adaptation [12–14], following ER. We then used a discovery-based multi-omics approach to build molecular signatures of ER in the BLA. Bioinformatics analyses of these signatures identified microglia (resident immune cells in the brain) [15–17] and the extracellular matrix (ECM) (scaffolding providing support for synaptic development and plasticity) [18–20] as consistently altered targets. Both microglia and ECM have complex relationships with stress, making them interesting candidates here [18, 19, 21, 22]. Follow up molecular and behavioral studies revealed how ER-induced changes in these candidate mechanisms impact BLA plasticity and BLA-dependent behaviors. We then demonstrated that these loss-like phenotypes could be rescued by enzymatically depleting the BLA ECM during the removal period. Taken together, this information-rich dataset provides unique insight into a role of the ECM in driving diverse neural and behavioral dysfunctions consequent to loss in the amygdala.

## Methods

### Subjects

Eight-week-old adult male Sprague-Dawley rats were used for all experiments. All procedures were conducted in compliance with the National Institutes of Health Guidelines for the Care and Use of Animals and approved by the University of Cincinnati Institutional Animal Care and Use Committee.

### Enrichment removal protocol

**Fig. 1:**
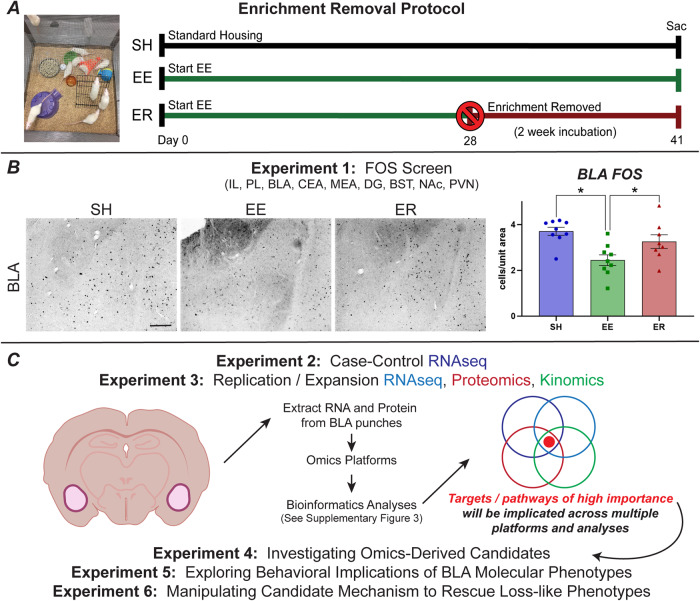
Experimental background and overview. **A** Example of environmental enrichment (EE), and timeline for standard enrichment removal (ER) protocol. **B** Representative images of the BLA from initial Fos IHC screen and quantification (SH *n* = 9, EE *n* = 9, ER *n* = 8; scale bar = 100 µm). **C** Outline for omics and follow-up studies. Experiments 2 and 3 utilized multi-omics in tandem with a number of complementary bioinformatics analyses to explore the molecular landscape of the BLA following ER. Experiments 4 and 5 further explored the candidate mechanisms that emerged from these analyses, as well as their role in ER behavioral phenotypes, to expand our understanding of novel mechanisms that underlie loss. Experiment 6 explores manipulating the molecular mechanisms identified here in an attempt to rescue the loss-like behavioral phenotypes generated by ER. * = *p* < 0.05.

Rats were randomly assigned to 3 housing conditions: standard housing (SH), environmental enrichment (EE), and enrichment removal (ER). SH consisted of either single-housing (Experiments 1–2) or pair-housing (Experiments 3–5) (Supplementary Table 1). Prior experiments suggest that single and pair-housed male controls are highly similar, and that social isolation or removal from social enrichment alone is insufficient to generate ER phenotypes [8, 9]. Enrichment housing consisted of 10 rats housed in a 1 m^3^ multi-level wire mesh cage with 5–6 toys and shelters that rotated weekly (Fig. 1A). ER rats were maintained in the same conditions as EE rats for 4 weeks. ER rats were then removed from EE and placed into 24-h single housing for the remainder of the experiment, with no further enrichment. These studies were primarily focused on ER effects, with SH serving as a procedural control and EE serving as the canonical experiential control [8].

### Experimental design

Six experiments were conducted for the present analyses. Note that ER rats consistently gained weight across experiments (Supplementary Fig. 1). All analyses were conducted by a blinded observer. For full experimental details, see supplementary methods.

Fos expression profiling was first used to identify stress-responsive brain regions that showed differential responsivity to EE and ER. Regions examined included the basolateral amygdala (BLA), medial amygdala (MEA), central amygdala (CEA), infralimbic prefrontal cortex (IL), prelimbic prefrontal cortex (PL), bed nucleus of the stria terminalis (BST), paraventricular nucleus(PVN), nucleus accumbens(NAc), and dentate gyrus (DG). These regions have known roles in stress responsivity, making them likely candidates for dysregulation by chronic stress [23–25]. Animals were sacrificed 90 min after forced swim, and immunohistochemistry was used to assess Fos activation to acute stress.

The BLA emerged as a region of interest. BLA micropunches were collected from SH, EE, and ER rats 2 weeks after removal. RNA was extracted and submitted for RNAseq. Our comprehensive bioinformatics pipeline, which included full transcriptome pathway analysis, targeted pathway analysis, and perturbagen analysis, (Supplementary Fig. 3) was used to assess EEvSH, ERvSH, and ERvEE molecular profiles.

A new cohort was generated to run parallel RNAseq, shotgun proteomics, and serine-threonine kinomics. This triple-omics approach not only served as a validation of Experiment 2, but also allowed us to examine ER-induced changes at multiple molecular levels. RNA and protein were extracted from BLA micropunches using our triple-prep protocol, and pooled samples were submitted to respective cores. Results were analyzed via our bioinformatics pipeline with the goal of identifying targets that replicated across cohorts, platforms, and analyses.

Immunohistochemistry was used to investigate targets implicated by the multi-omics, particularly BLA microglia and ECM. Microglial endpoints included area (total IBA area/microglia count), morphology (Imaris process analyses), and phagocytosis (CD68 expression/microglia). ECM endpoints included overall area (total WFA area) and characteristics relating to perineuronal nets (PNNs) decorating parvalbumin interneurons (PV) (PNN counts, PV synaptic inputs).

To better understand the impact of these molecular changes, we then expanded our behavioral profile of ER to behaviors known to centrally involve the BLA. Behavioral testing was run in two cohorts and included passive avoidance, social threat, acoustic startle, and fear conditioning. Brains were collected to assess Fos activation of BLA PV cells following fear conditioning reinstatement.

Finally, we utilized Chondroitinase ABC (ChABC), a bacterial enzyme which digests ECM, to deplete BLA PNNs during removal and test if we could block the development of loss-like behaviors. EE was chosen as the control group because it represents the more informative control for loss. EE and ER rats received injections of ChABC or vehicle into the BLA at the start of the removal period, and were then subjected to passive avoidance, acoustic startle, and forced swim testing. Brains were collected to verify injection sites and ECM depletion. See supplementary methods for full methodological details.

### Statistics

Normality and variance were assessed using GraphPad Prism 9. Molecular and behavioral endpoints were analyzed by one-way ANOVAs and two-way repeated measures ANOVAs. Kruskal-Wallis non-parametric tests were used when necessary. Pearson correlations were used where appropriate (GraphPad Prism 9). Based a priori on prior studies, outliers were determined by values that fall outside the mean ± 1.96 times the standard deviation [8, 9]. All post hoc testing utilized Tukey tests (Supplementary Table 8).

## Results

### Experiment 1: BLA exhibits differential Fos activation following ER

We initially ran a post-forced swim Fos screen to identify regions putatively contributing to loss phenotypes (Fig. 1A). The most notable pattern of Fos expression was in the BLA, where ER produced a pronounced increase in the number of Fos reactive neurons relative to continuously-enriched controls (Fig. 1B; Supplementary Fig. 2). The IL also demonstrated differential Fos expression, albeit weaker. The other regions did not show differential Fos responsivity. Given the magnitude of the changes and the established role of the BLA in stress processing and adaptation [12, 13], this region was selected for further analysis.

### Experiment 2: RNAseq points to immune and matrix alterations following ER

We designed a series of hypothesis-generating experiments to uncover potential mechanisms underlying the effects of ER in the BLA (Fig. 1C). Analyzing data as complex as transcriptomic signatures with a singular platform is inherently biased and could miss novel information that could be gleaned from more comprehensive analyses. Here we leveraged a pipeline that covers multiple dimensions of transcriptomic analyses and thus lends more confidence to interpretation of gene expression patterns (Supplementary Fig. 3).

Micropunches of the BLA were collected [26], RNA was extracted, and samples were submitted for case-control RNAseq. Transcriptomic data was generated for SH, EE, and ER rats, yielding signatures for three contrasts: EEvSH, ERvSH, and ERvEE (Supplementary Table 2). EEvSH represents changes generated by the experience of enrichment, ERvSH represents changes generated by the full experience of enrichment and removal, and ERvEE represents changes generated specifically by removal. Both ERvSH and ERvEE contrasts are important to understanding ER phenotypes, as comparison to EE (canonical experiential control group) indicates how loss specifically impacts the BLA and comparison to SH (procedural control group) indicates how those changes compare to rats housed in unstimulated conditions.

**Fig. 2:**
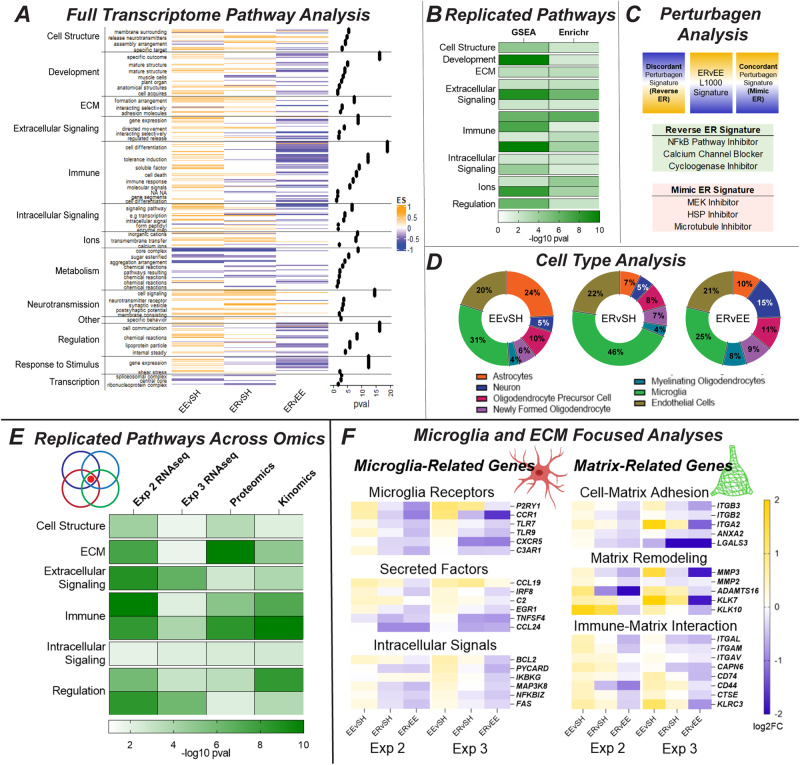
Multi-omics indicates ER alterations to microglia and the extracellular matrix. **A** Results from Experiment 2 (*n* = 6/group) (Supplementary Table 4). The first analysis utilized GSEA to conduct full transcriptome pathway analysis. Significant pathways (*p* < 0.05) were condensed based on semantic similarity using Pathway Hunter (small left labels, and these categories were further condensed into functional themes based on a priori knowledge (leftmost labels)). Enrichment scores for individual pathways are represented by the heatmap (with EEvSH on the left, ERvSH in the middle, and ERvEE on the right), and the degree of each category’s enrichment is represented by the *p* value dots on the right. The enrichment scores show how much (magnitude) and in what direction (yellow is up, blue is down) a theme was changed by the model, while the *p* values show the contribution of each category to these changes. **B** The second analysis utilized Enrichr to conduct targeted pathway analysis and generated a similar set of results. Themes that replicated between these two pathway analyses are shown in the replicated pathways heatmap, with darker green indicating more involvement. **C** The third analysis utilized iLINCS to look for compounds that would replicate (concordant) or reverse (discordant) the present phenotypes. **D** We also used the leading-edge genes from GSEA to conduct cell type analysis. Collectively, these pipeline results start to implicate microglia and the extracellular matrix (ECM) in ER. **E** Similar analyses were run on the data from Experiment 3 (*n* = 10/group pooled). The RNAseq results are presented in Supplementary Fig. 5, while the proteomics and kinomics results are presented in Supplementary Fig. 6. Those results are summarized and compared to the above results here, and they provide more support for microglia and the ECM. This replication across cohorts, omics platforms, and analyses gives us higher confidence in these targets’ involvement. **F** Hypothesis-driven analyses of microglia and ECM-related genes further implicates both at multiple levels and points to a pattern of upregulation in EE (yellow), followed by downregulation in ER (blue).

The first analysis used gene set enrichment analysis (GSEA) [27] to broadly assess gene expression across the full transcriptome. Significantly enriched gene ontology pathways were identified for each contrast and organized into functional categories using PathwayHunter [28] (see methods; Fig. 2A, small labels; Supplementary Fig. 4A; Supplementary Table 4). These categories were further condensed based on a priori knowledge (Fig. 2A, large labels), and the strength and direction of changes in these categories was determined based on hypergeometric overlap (Fig. 2A, −log10 *p* values) and GSEA enrichment score (Fig. 2A, colors), respectively. Many of the observed pathways were upregulated in EE (EEvSH, yellow) and downregulated in ER (ERvEE, blue). Several categories were altered in ERvSH, indicating that ER is not merely a return to SH, but a dysregulation within these processes. Strongly implicated functional categories included *immune, extracellular signaling, development, regulation, response to stimulus*, and *cell structure*.

The second analysis focused on the strongest differential expression. The top 100 upregulated and top 100 downregulated genes (by fold change) were determined for each contrast and entered into Enrichr for pathway analysis [29]. The results were condensed using PathwayHunter and yielded a similar pattern of upregulation in EE and downregulation in ER (Supplementary Fig. 4B; Supplementary Table 4). While fewer overall pathways were found in this dataset, we again observed strong involvement of *immune, extracellular signaling*, and *regulation*.

Directly comparing the functional categories implicated by both full transcriptome and targeted pathway analysis emphasizes the consistencies between the two approaches (Fig. 2B, Supplementary Fig. 4C; Supplementary Table 4). Specifically, themes observed in both analyses were: *extracellular matrix, extracellular signaling, immune, cell structure, development, intracellular signaling, ions*, and *regulation*.

Our third analysis utilized iLINCS (integrated network-based cellular signatures), a publicly available database curating transcriptomic signatures for thousands of drugs and compounds (aka perturbagens) [30]. iLINCS’s signatures are based on the L1000, a set of 978 genes measured in cell lines treated with these perturbagens. L1000 signatures were extracted for each contrast and uploaded to iLINCS. Similar perturbagen signatures were “concordant,” meaning that the compounds would be expected to recapitulate the experimental phenotype. On the other hand, different perturbagen signatures were “discordant,” meaning that the compounds would be expected to reverse (aka treat) the experimental phenotype (Fig. 2C). Findings here lend additional support for our pathway results. Discordant perturbagens involved the immune system (*NFkB and cyclooxygenase inhibitors*) and signaling (*calcium channel blocker*), while concordant perturbagens involved cell structure (*microtubule inhibitor*), response to stimulus (*HSP inhibitor*), and signaling *(MEK inhibitor*) (Fig. 2C). Looking more broadly at mechanism of action (MOA) revealed that the strongest discordant perturbagens would impact the immune system, while the strongest concordant perturbagens would impact kinase signaling, suggesting specific signaling mechanisms that may be involved in loss (Supplementary Fig. 4D; Supplementary Table 5).

We then used leading-edge genes obtained from GSEA (i.e., genes that drove the enrichment of significant pathways), to determine the contribution of individual cell types to the pathway results. The top 100 upregulated and top 100 downregulated leading-edge genes (by number of pathways) were uploaded into Kaleidoscope and their typical cell-type enrichment was determined using the “Brain RNA-Seq” module [31]. Proportions of this enrichment were determined to reveal the relative contribution of different cell types. Microglia exhibited the biggest contribution across all contrasts (Fig. 2D; Supplementary Table 6), followed by endothelial cells, astrocytes, and oligodendrocytes. Neuronal involvement was relatively small, supporting the notion that loss mechanisms favor the brain’s support systems over direct effects on neuronal circuitry itself.

### Experiment 3: Multi-omics supports microglia and extracellular matrix phenotypes

We next sought to validate and expand our ER signatures. This experiment provided 1) replication RNAseq and 2) omics data from other platforms that could potentially reveal whether changes observed at the RNA level survived to the protein (shotgun proteomics) and activity (serine-threonine kinomics) levels. BLA micropunches from a new cohort were subjected to our “triple prep” protocol (see methods) allowing for simultaneous RNA and protein extraction. This preparation ensures that changes observed on multiple platforms were real and not an artifact of tissue heterogeneity. Extracted samples were then processed for parallel RNAseq, proteomics, and kinomics. Samples were pooled within groups.

The RNAseq was analyzed in the same manner described above (Supplementary Table 2). While individual pathways showed some variation (Supplementary Fig. 5A, B; Supplementary Table 4), the functional categories associated with ER were similar to those observed in Experiment 2 (Supplementary Fig. 5C; Supplementary Table 4). Similar patterns of upregulation in EE and downregulation/dysregulation in ER were observed, as well as strong involvement of the immune system, ECM, and regulation across full and targeted pathway analyses. Signature analysis again implicated the immune system and kinases (Supplementary Fig. 5D; Supplementary Table 5), while cell type analysis again pointed to microglia as the most involved cell type (Supplementary Fig. 5E; Supplementary Table 6).

This experiment also offered insight into ER-related changes in protein and activity. EEvSH, ERvSH, and ERvEE signatures were generated for each platform (Supplementary Table 3), and targeted pathway analysis was run in Enrichr. The targeted method was selected over the full transcriptome method because neither of these platforms yields data from enough targets to properly conduct the latter. Proteomics signatures were based on the top 100 up- and down-regulated peptides (by fold change), while kinomics signatures were based on differentially phosphorylated peptides (FC > 1.2) [32].

The proteomics exhibited more upregulation across the different contrasts but involved similar themes as the RNAseq, including strong involvement of the ECM, immune system, and cell structure (Supplementary Fig. 6A; Supplementary Table 4). The kinomics yielded a pattern similar to the transcriptomics, with upregulation in EE followed by downregulation/dysregulation in ER, and implicated the immune system, cell cycle, regulation, and signaling (Supplementary Fig. 6B; Supplementary Table 4). Upstream kinase analysis was also performed using KRSA, pointing to several kinases that may be implicated in ER (Supplementary Fig. 6C; Supplementary Table 3) [32].

The multi-omics demonstrates that EE and ER had major effects on the BLA’s molecular landscape, and that ER triggered changes differing from both EE and SH. While specific signatures from each omics platform varied to a degree, commonly implicated RNAseq themes were largely supported by both the proteomics and kinomics (Supplementary Fig. 6D; Supplementary Table 4). Both indicated involvement of the immune system and ECM, indicating that these changes survive across multiple molecular domains of the BLA following ER (Fig. 2E; Supplementary Table 4).

Given the emergent roles of microglia and ECM in our hypothesis-free analyses, we went back into the omics datasets with this notion in mind. Looking specifically at genes associated with microglia, ECM, and the interaction of the two [15, 33, 34], we observed strong patterns of upregulation in EE and downregulation in ER, suggesting a loss of function with microglia activity and ECM regulation following ER (Fig. 2F; Supplementary Table 7). Genes related to microglia receptors, secreted factors, and intracellular signals suggest that microglia homeostatic functions related to surveying and interacting with their surroundings may be attenuated [15–17, 21]. Genes related to cell-matrix adhesion and matrix remodeling suggest that the ECM may not be assembling or degrading in the expected manner [18, 19, 35]. Genes related to immune-matrix interaction suggest that these alterations could be linked [33, 36, 37].

These gene responses further demonstrate that ER is distinct from SH. ER causes some genes to remain elevated at EE levels (e.g., CCL19, KLK7, ITGAV), some to return to SH levels (e.g., TLR7, ITGB3, CD74), and some to drop below SH levels (e.g., CCR1, C3AR1, LGALS3). ER also impacted some genes that were unaffected by EE (e.g., CCL24, TNFSF4, CXCR5). The differences between SH, EE, and ER show that ER is not merely a reversal of EE, but a unique condition that generates its own molecular state.

### Experiment 4: Validating and expanding omics-derived candidate mechanisms

A new cohort of ER rats was generated to further explore these mechanisms. Rats were perfused, and immunohistochemistry (IHC) was used to validate and elaborate upon microglia and ECM phenotypes. See supplement for full IHC protocols and antibody information.

#### BLA microglia are smaller, less complex, and less phagocytic following ER

**Fig. 3:**
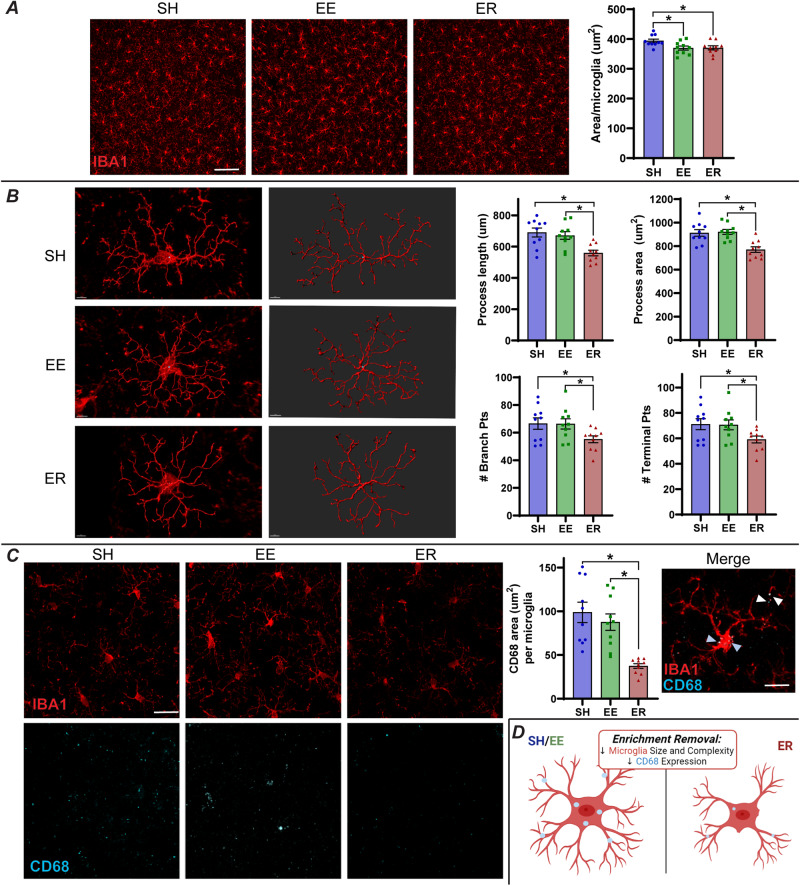
Microglia are smaller, less complex, and less phagocytic following ER. **A** Initial analysis of BLA microglia (labeled with IBA1) suggested that microglia are smaller in EE and ER (*n* = 10/group; scale bar = 100 µm). **B** A more robust analysis of microglia morphology was conducted using the filament tracer tool in Imaris (*n* = 10/group; *N* = 8 microglia/animal) and revealed that, in ER alone, microglia are smaller and less complex. **C** ER rats express significantly less CD68 than SH or EE rats, suggesting that they are less phagocytic (*n* = 10/group; scale bar = 10 µm). Merge shows localization of CD68 to microglia (scale bar = 3 µm). White arrows indicate CD68 in microglia processes, while blue arrows indicate CD68 in microglia soma. No WFA inclusions were observed within CD68. **D** Summary of ER effects on microglia phenotypes. * = *p* < 0.05.

IBA1-immunoreactive area/microglia was reduced in both EE and ER relative to SH in the BLA (Fig. 3A), with no changes in cell counts, labeling intensity, or soma size (Supplementary Fig. 7A–C). Further morphological analysis in Imaris revealed that the ER group exhibited shorter and smaller processes, fewer branch points, and fewer terminal points (Fig. 3B). A robust decrease of CD68 area/microglia was observed in the ER group, suggesting that ER microglia are less phagocytic (Fig. 3C). These results align well with the omics data, suggesting ER microglia exhibit less surveillance of and interaction with their surroundings (Fig. 3D) [15–17].

#### ER increases BLA ECM and Perineuronal Nets

**Fig. 4:**
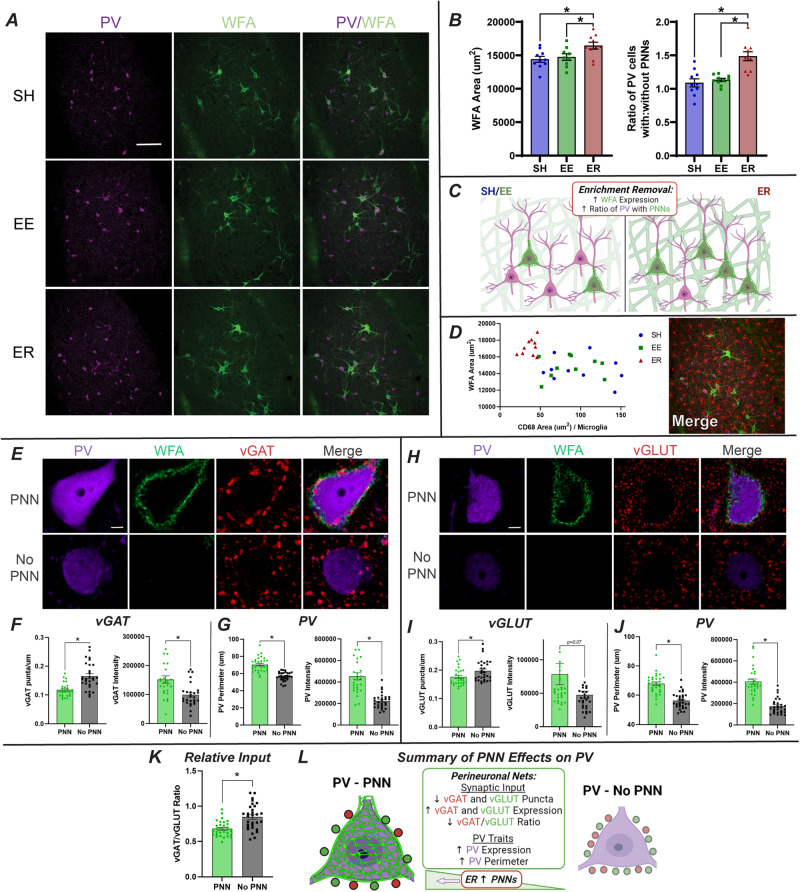
ER increases BLA ECM and PNNs on PV interneurons, altering PV synaptic inputs and phenotypic characteristics. **A** Representative images of PV and WFA staining in the BLA (SH *n* = 10, EE *n* = 9, ER *n* = 10; scale bar = 100 µm). **B** ER increases WFA area (µm^2^) and the ratio of PV cells decorated with PNNs (*n* = 10/group). **C** Summary of ER effects on matrix phenotypes. **D** A significant correlation between WFA and CD68 suggests that microglia may be digesting the matrix less in ER, contributing to the present increases. Image shows IBA1/WFA/PV merge in the BLA (*n* = 10/group). **E** Representative images of vGAT puncta surrounding PV cells with and without PNNs (*n* = 10/group). **F** PNNs decrease vGAT puncta/µm (PNN *N* = 30, No PNN *N* = 30 collapsed) and increase vGAT expression (PNN *N* = 29, No PNN *N* = 30) in those puncta. **G** PV cells with PNNs are larger (PNN *N* = 30, No PNN *N* = 30) and express more PV (PNN *N* = 29, No PNN *N* = 29). **H** Representative images of vGLUT puncta surrounding PV cells with and without PNNs (*n* = 10/group). (I) PNNs decrease vGLUT puncta/µm (PNN *N* = 30, No PNN *N* = 30 collapsed) and increase vGLUT expression (PNN *N* = 30, No PNN *N* = 29) in those puncta. **J** PV cells with PNNs are larger (PNN *N* = 30, No PNN *N* = 30) and express more PV (PNN *N* = 30, No PNN *N* = 30). **K** PNNs decrease the ratio of vGAT/vGLUT (PNN *N* = 30, No PNN N = 30). **L** Summary of PNN effects on PV inputs and phenotypes. Given that ER increases PNNs, one would expect a shift in PV phenotypes in ER similar to those seen in PNNs here. * = *p* < 0.05.

We used histochemistry to assess BLA expression of WFA (Wisteria Floribunda Agglutinin), which labels the ECM’s CSPGs (Chondroitin Sulfate Proteoglycans) [19, 35]. CSPGs are a critical component of ECM in general and in perineuronal nets (PNNs), mesh-like ECM formations that surround neurons and help to regulate cell physiology. PNNs play a large role in neuroplasticity [18, 20, 38], making them a potentially intriguing target in ER. As PNNs primarily surround inhibitory parvalbumin (PV) interneurons [19, 35], we co-labeled tissue for PV. WFA staining area was increased in ER rats relative to both EE and SH controls, as was the ratio of PV-immunoreactive cells decorated with PNNs (Fig. 4A–C; Supplementary Fig. 7D). The number of PV cells did not change between groups (Supplementary Fig. 7E).

#### Connecting microglia and ECM

Our omics results suggested that the changes in ER microglia and ECM could be related. Microglia do play a role in digesting ECM, through mechanisms like integrin receptor signaling, matrix metalloprotease secretion, and phagocytosis [18, 34, 37, 39]. Here, we observed a significant correlation between microglial CD68 and WFA area (Fig. 4D). While just a correlation, ER animals cluster together, demonstrating low CD68 and high WFA, suggesting that the loss of function in microglia could contribute to the buildup of ECM in ER.

#### PNNs alter PV synaptic inputs and characteristics

PNNs help regulate PV cell physiology [19, 20, 35], so the present ER-related increase in PNNs could impact the BLA’s inhibitory tone via changes to PV characteristics [13, 40], an effect that could substantially impact ER behaviors. One way PNNs regulate PV is by altering synaptic inputs, acting as a physical guide for appositions onto PV [18, 38]. Therefore, we tested whether the presence of PNNs alters inhibitory (vGAT, Fig. 4E) and excitatory (vGLUT, Fig. 4H) synaptic inputs onto BLA PV cells. To eliminate sampling bias, we assessed vGAT and vGLUT terminals on equal numbers of PV cells with (*n* = 4) and without (*n* = 4) PNNs in each animal. This approach focused on the question of PNN effects, rather than group effects, on PV inputs, so all groups were collapsed into PNN vs no PNN comparisons.

Both vGAT (Fig. 4F) and vGLUT (Fig. 4I) puncta were decreased on PV cells with PNNs. However, puncta on PNN-bearing cells were significantly more intensely labeled, suggesting increased capacity for packaging of GABA and glutamate, respectively. Thus, these connections may be more efficient, favoring fewer, mature puncta over more numerous, but less established puncta [41, 42]. These effects were more robust in vGAT than vGLUT, a change made evident by the decreased vGAT/vGLUT ratio in PV cells with PNNs (Fig. 4K). A decreased vGAT/vGLUT ratio indicates less relative inhibitory input onto these cells, suggesting that PNNs may allow for increased PV activity [43, 44]. Differences in the PV cells themselves were observed, with PNN-PV cells expressing more PV and having larger perimeters (Fig. 4G,J). These changes are indicative of more mature PV neurons with an increased calcium buffering capacity [45, 46], another sign of potentially more active PV cells with PNNs.

While this analysis focused on PNN effects precluded our ability to directly observe group effects (Supplementary Fig. 7F,G), it is important to note that PNN-positive PV neurons were increased in ER relative to both control groups (Fig. 4B). Thus, the impacts on synaptic efficacy and cellular maturity imparted by PNNs would disproportionately affect BLA PV neurons following ER (Fig. 4L).

### Experiment 5: Expanding BLA behaviors and their link to PNNs

We next explored the impact of ER on behaviors specifically linked to the BLA. We generated two new ER cohorts and conducted a series of behavioral tasks that are known for BLA involvement, including passive avoidance, social threat, acoustic startle, and cued fear conditioning [12, 13, 47–49].

**Fig. 5:**
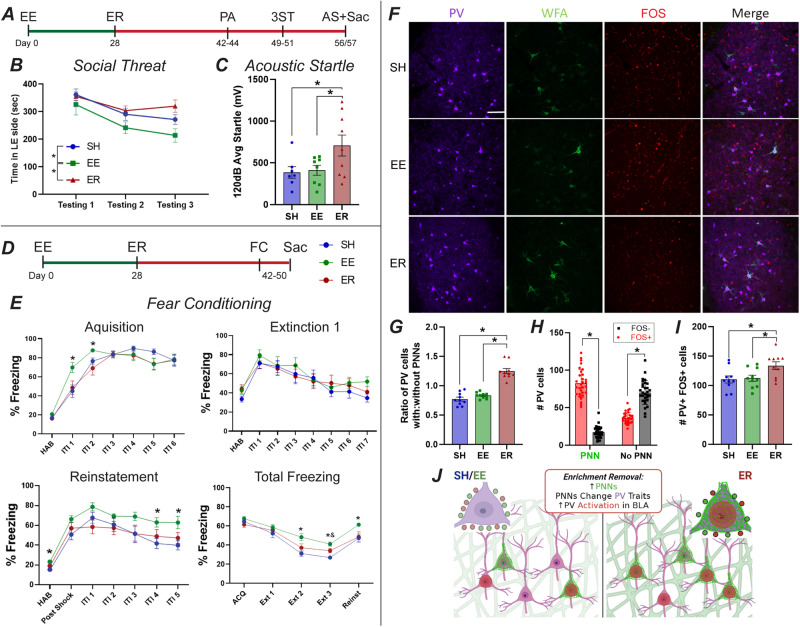
ER impairs BLA-related behaviors and activates PV interneurons. **A** Timeline for passive avoidance (PA), three chamber social threat (3ST), and acoustic startle (AS) cohort. **B** Relative to EE rats, ER rats spend more time in the “threatening” side that previously housed an aggressive Long Evans rat (SH *n* = 7, EE *n* = 9, ER *n* = 9). **C** ER rats show enhanced startle response (SH *n* = 7, EE *n* = 8, ER *n* = 9). **D** Timeline for fear conditioning cohort. **E** Fear conditioning results (*n* = 10/group). Relative to EE, ER rats exhibited delayed acquisition, faster extinction, and lower reinstatement. The first three panels show % freezing on individual days, while the last shows total % freezing across all 5 days. **F** Representative images of WFA/PV/FOS IHC (*n* = 10/group). **G** Increased ratio of PV with PNNs in ER replicated in this cohort (*n* = 10/group). **H** PV cells with PNNs were more likely to be FOS+ than PV cells without PNNs (*n* = 10/group). **I** Together these differences resulted in more PV + FOS+ cells in ER, indicating increased PV response to stress following ER (*n* = 10/group). **J** Summary of FOS results and their connection to the above results regarding PNNs and PV. * = *p* < 0.05 for EE vs ER/SH. & = *p* < 0.05 for ER vs SH.

Two weeks after removal, rats received passive avoidance testing, followed by exposure to social threat in the three-chamber apparatus and subsequent assessment of acoustic startle (Fig. 5A). No differences were detected between groups in the passive avoidance test [12, 47] (Supplementary Fig. 8A). In the social threat test, ER rats spent more time in the chamber where they had previously encountered a Long Evans retired breeder than EE rats (Fig. 5B; Supplementary Fig. 8B), suggesting reduced fear memory relative to experienced animals in a social context [48]. Finally, startle responses were enhanced in ER animals (known to be regulated by BLA) [13, 49] (Fig. 5C; Supplementary Fig. 8C).

A second cohort was tested for cued fear conditioning and freezing responses (Fig. 5D). ER rats acquired fear conditioning more slowly and extinguished more rapidly than EE rats, while also exhibiting reduced fear reinstatement (Fig. 5E; Supplementary Fig. 8D). These results suggests that ER impairs fear learning and fear recall relative to EE in this more complex task [12, 47]. There was no major difference evident between SH and ER groups, suggesting that ER actions are specific to loss of EE.

Taken together, these results suggest that ER generates a unique behavioral profile that is related to impaired fear recall and adaptability. Both of these behavioral effects are consistent with a BLA that has attenuated plasticity and heightened inhibition [12, 13], tying these phenotypes back to the molecular effects observed above.

To test possible connections of ER-related behavioral changes to BLA activation, we assessed Fos induction following reinstatement testing (Fig. 5D,F). The finding of increased PNNs on PV cells in ER rats replicated (Fig. 5G), and expression of Fos was disproportionately enhanced in PV neurons surrounded by PNNs (Fig. 5H). Importantly, Fos activation of PV neurons was increased in ER rats relative to both control groups (Fig. 5I), further supporting the notion of increased PV-mediated inhibition in the BLA following ER (Fig. 5J).

### Experiment 6: Testing the necessity of BLA PNNs in loss-like behaviors

Lastly, we sought to establish a causal link between BLA PNNs and ER. Utilizing the enzyme ChABC which digests ECM and PNNs [20, 34], we investigated if blocking the increase in PNNs that occurs during ER could also block the development of loss-like behaviors. ChABC rapidly digests PNNs upon injection (~90% depletion), and PNNs then gradually repopulate over the next few months. In our pilot, PNNs were still ~60% depleted 3 weeks after injection (Supplementary Fig. 9A).

**Fig. 6:**
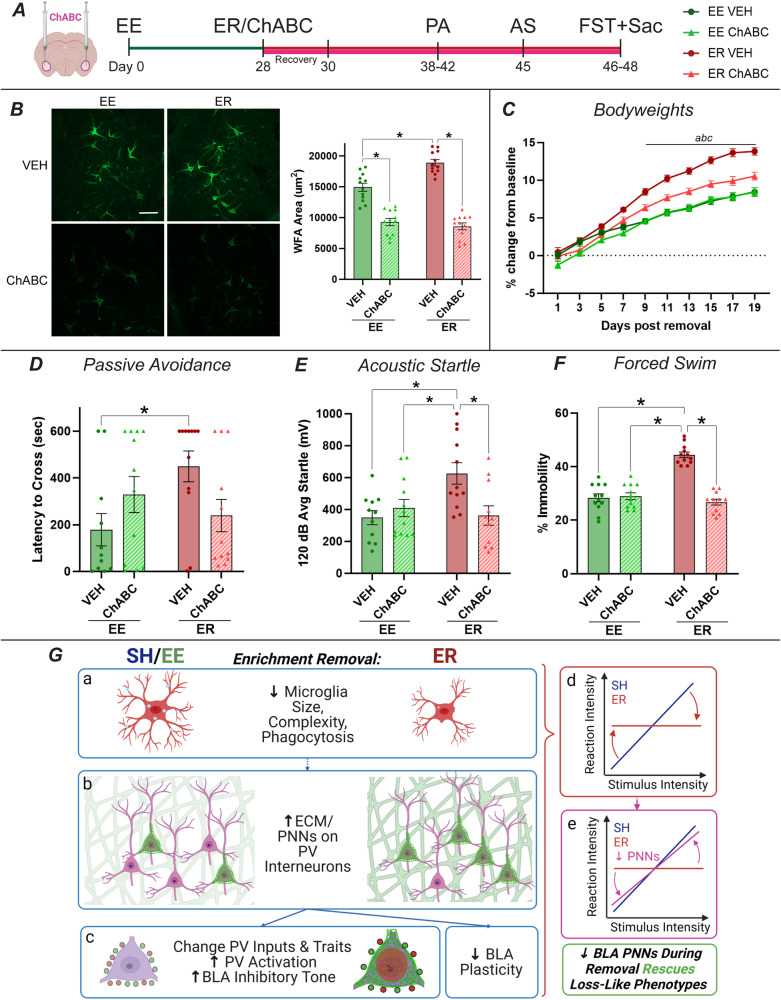
Depleting BLA PNNs rescues ER behavioral phenotypes. **A** Timeline. Chondroitinase ABC (ChABC) was injected into the BLA at the time of removal to deplete PNNs (*n* = 12/group). Rats were then subjected to passive avoidance (PA), acoustic startle (AS), and forced swim testing (FST). This study was a 2 × 2 design with housing (EE or ER) and treatment (ChABC or VEH) as factors. **B** Validation of ChABC effects on PNNs (*n* = 12/group). ER again increased BLA WFA area, while ChABC successfully reduced PNNs in all animals. **C** Post-removal bodyweights show a blunting of ER effects in ER ChABC rats (*n* = 12/group). **D** ER VEH rats exhibited increased fear recall in passive avoidance testing relative to EE VEH rats, an effect that was blocked in ER ChABC rats (EE VEH *n* = 12, EE ChABC *n* = 12, ER VEH *n* = 11, ER ChABC *n* = 12). **E** Similarly, ER VEH rats showed a hyperresponsiveness to acoustic startle that was blocked by ChABC (EE VEH *n* = 12, EE ChABC *n* = 12, ER VEH *n* = 12, ER ChABC *n* = 11). **F** In the forced swim test, ER VEH rats exhibited increased immobility, while ER ChABC did not (*n* = 12/group). **G** Overall summary of the relationship between BLA microglia, ECM, and loss-like behaviors. Collectively, the omics, molecular, and behavioral results presented here suggest the following: (a) ER leads to a loss of function in microglia that decreases their size, complexity, and phagocytic activity. (b) ER also increases ECM/PNNs in the BLA. These two findings may or may not be connected. (c) Increased PNNs influence PV interneurons, leading to increased PV activation and inhibitory tone in the BLA. Increased ECM in general is also expected to decreased plasticity within the BLA. (d) Together, these molecular changes appear to interfere with the BLA’s role in salience evaluation, uncoupling rats’ reactions from the intensity of the stressors and causing them to react too much or too little to various stimuli. (e) This effect can be largely rescued by depleting BLA PNNs, blocking ER molecular effects to restore the BLA’s salience evaluation capabilities. Ultimately, these complex mechanisms hold multiple similarities to loss in humans and represent several novel targets that could be used in developing therapeutics for people suffering from loss. * = *p* < 0.05. a = *p* < 0.05 for ER VEH vs EE VEH. b = *p* < 0.05 for ER VEH vs ER ChABC. c = *p* < 0.05 for EE VEH vs ER ChABC.

With this timeframe established, EE and ER rats received bilateral injections of either ChABC or VEH into the BLA at the start of the removal period (Fig. 6A) and received passive avoidance, acoustic startle, and forced swim testing within 3 weeks. Within the BLA, ER again increased, while ChABC significantly reduced, WFA area (Fig. 6B). The complex design of this study limited the number of control groups possible. EE was selected over SH because it is the canonical experiential control for loss-specific effects. The ultimate goal here is to return ER animals to the positive stimulated state of EE. We also selected behavioral endpoints that did not differ amongst SH and EE groups to mitigate the loss of the SH group.

One of the most established ER phenotypes is weight gain (Supplementary Fig. 1). ER rats that received BLA ChABC exhibited a blunted weight gain compared to ER VEH rats (Fig. 6C). Behaviorally, ER VEH rats also exhibited multiple phenotypes that were blocked in ER ChABC rats. Latency to cross to the shock-conditioned side in passive avoidance was increased in ER VEH rats (Fig. 6D; Supplementary Fig. 9B), the startle response was enhanced (Fig. 6E; Supplementary Fig. 9C), and immobility was increased in the forced swim test [50] (Fig. 6F; Supplementary Fig. 9D). In each case, ChABC was able to block the effects of ER, returning ER ChABC rats to EE VEH levels. Taken together, these results suggest that ChABC rescues multiple ER phenotypes and supports the necessity of BLA PNN accumulation in ER behavioral phenotypes.

## Discussion

Psychological loss impacts most people at some point in their lives; however, the mechanisms underlying the experience of loss are poorly understood [1–3]. Here, we used enrichment removal (ER) in tandem with a series of comprehensive multi-omics, molecular, and behavioral approaches to investigate these mechanisms in the basolateral amygdala (BLA), as well as their subsequent functional consequences that contribute to loss-like phenotypes.

We first demonstrated that the BLA exhibited the strongest differential activation following ER. Then, multi-omics, spanning multiple cohorts, platforms, and analyses, indicated that BLA microglia and extracellular matrix (ECM) were dysregulated by ER. These relatively unexpected targets first emerged from hypothesis-free analyses, demonstrating the strength of our approach for identifying novel targets. The consistent implication of these targets across multiple levels affords us higher confidence that the changes are important to loss physiology. Further investigation supported these omics-derived hypotheses and revealed how ER dysregulates BLA microglia and ECM. ER decreased the size, complexity, and phagocytic activity of BLA microglia, traits which suggest that ER microglia have an impaired capacity to surveille and interact with their surroundings (Fig. 6Ga). While most studies assess conditions with overly active microglia and neuroinflammation, a loss of function in microglia can also be detrimental, as microglial monitoring and regulation of neurons and the microenvironment is critical to maintaining homeostasis. Indeed, depleting microglia can cause cognitive, social, and motor impairments [15–17, 21]. Thus, the apparent decrease in microglia surveillance and phagocytosis following ER could attenuate the functional capacity of the BLA.

ER-associated increases in ECM expression and organization (PNNs decorating PV interneurons) are likely indicative of decreased plasticity, as PNNs form a physical barrier to new connections, making decorated cells less able to adapt (Fig. 6Gb) [19, 35]. Furthermore, we observed several PNN-associated changes to PV interneurons indicative of greater synaptic efficacy, less relative inhibition, and increased PV activity (Fig. 6Gc). These findings align well the known roles of PNNs in protection from reactive oxygen species, participation in ion buffering, and organization of synaptic inputs [18, 38]. Depleting PNNs is known to decrease PV activity and maturity [44, 51], supporting the conclusion that PV cells with PNNs are more active. Indeed, PV cells exhibited greater Fos responses to reinstatement in ER rats, suggesting that the BLA has greater inhibitory tone following ER. A less plastic, more inhibited BLA supports the possibility of attenuated functional capacity. Future studies will be required to determine how changes in PV activity in PNN-bearing cells affect output and oscillatory activity within the BLA. Finally, the correlation between microglial CD68 and WFA area suggests that these phenotypes may be linked. Microglia depletion can increase the amount of ECM [37, 39], so it is possible that the decreased microglia phagocytic marker seen here could lead to increased ECM deposition [34, 37, 39] and a shift towards the PV phenotypes that accompany PNNs. Future studies will be needed to further evaluate the relationship between changes in microglial function and ECM hypertrophy.

The functional consequences of these molecular changes in the BLA could contribute to loss-like behavioral phenotypes. Generally speaking, the BLA acts as an important neural node responsible for selection of appropriate behavioral responses to stimuli. It performs this role by gathering polysensory and associational information from numerous regions, gauging the salience and valence of those inputs, and signaling downstream regions responsible for appropriate responses. The BLA is key to evaluating both of these factors, and flexibility within the BLA is required for rapidly evaluating multiple inputs and switching responses over time [12–14, 52, 53]. Thus, decreased plasticity and increased inhibition following ER would be expected to interfere with its capacity to evaluate and select the appropriate response to various stimuli. ER rats showed exaggerated responses to some stimuli (startle) while showing blunted responses to others (fear conditioning), suggesting impaired evaluation of salience (Fig. 6Gd) [12–14]. For example, startle responses may be enhanced by BLA inhibition, losing the ability to appropriately gate responses to the relatively low salience threat. In contrast, responses to high salience threats requiring freezing or social avoidance are attenuated. Our data align with previous studies noting that depletion of BLA ECM increased behavioral flexibility and adaptability [20, 34]. We functionally tested this relationship by depleting BLA PNNs during the removal period. This manipulation successfully rescued multiple ER physiological and behavioral phenotypes, blunting weight gain and blocking enhanced avoidance, startle, and passive coping. These findings support the necessity of BLA PNN accumulation in driving behavior following ER, suggesting an intriguing target for ameliorating loss. This suggests that blocking the increase in PNNs that occurs during ER can, at least partly, restore an animal’s capacity for salience evaluation, possibly via increased plasticity and dampened inhibition (Fig. 6Ge). Interestingly, ChABC had minimal effects in EE rats, suggesting that there is a unique interaction between the state of loss and PNN depletion.

We did not elect to test females in this paradigm due to fundamental differences occurring within preliminary RNAseq data that preclude this candidate mechanism for loss pathology. Where male RNAseq pathway analysis implicated BLA immune and extracellular pathways, similar analyses in females did not support a role for these candidates in ER. Instead, females demonstrated greater dysregulation in metabolic, developmental, and hormonal pathways (Supplementary Fig. 10). Taking a similar approach to studying female loss mechanisms is a logical next step, since they manifest significant (albeit different) behavioral responses to loss [9].

We identified the EE group as the canonical control for these studies, with the SH group included as a procedural control. The EE group shares the experiential aspect that is removed in the ER paradigm. Our prior work in males indicates that neither social isolation nor removal from social enrichment alone replicates an ER phenotype [8, 9]. Here, our omics data indicate that ER does not simply reverse endpoints to SH levels, indicating that the latter does not represent a “baseline condition” to which the organism returns. Studies in mice indicate impairments in socially isolated controls, using adolescent BALB/c mice and a distinct removal protocol [54]. However, a similar study performed in Long-Evans rats yielded results more in-line with those seen here [55]. While social isolation surely contributes to ER phenotypes, it is the experience of having had EE and lost it that forms the basis for the present study. Moreover, we observed multiple ER behavioral and molecular phenotypes that differed from those of both SH and EE controls, suggesting that loss is a unique state dealing with withdrawal of multiple positive stimuli and that the microglia/ECM phenotypes examined here play a unique role in loss. That being said, future studies will be needed to disentangle the differing contributions of removal of the different components of EE (i.e., social, physical, cognitive) to these complex ER phenotypes and mechanisms.

Humans suffering from loss often exhibit emotional blunting and impaired adaptability [1, 2, 6]. This emotional rigidity aligns well with the mechanisms proposed here, namely that loss of a motivationally significant stimulus impairs an individual’s salience evaluation, resulting in a mismatch between stimuli and response. These experiments put forth BLA PNNs an intriguing target for ameliorating loss symptoms in humans. In addition, symptoms of atypical depression (often precipitated by loss) are associated with decreased BLA activity and responsiveness [1, 56], suggesting that the local loss-related neurobiological changes in microglia, ECM, and PV interneurons observed here could have broader implications for a range of mood disorders. Further understanding the neurobiology of loss will be essential for development of both behavioral and pharmaceutical intervention approaches to limit its nearly universal impact on health and well-being.

### Supplementary information

Supplementary Information Key and Supplementary Figures

Supplementary Table 1

Supplementary Table 2

Supplementary Table 3

Supplementary Table 4

Supplementary Table 5

Supplementary Table 6

Supplementary Table 7

Supplementary Table 8

## Data Availability

RNAseq data are available at Gene Expression Omnibus under accession number GSE208029. All other data are available in the main text or supplementary materials.

## Code availability

Codes which have been previously published are cited in the manuscript. Additional in-house codes used to analyze multi-omics results can be found on GitHub at https://github.com/CogDisResLab/BioPathNet.
