# Supplementary material for: Molecular neurobiology of loss: a role for basolateral amygdala extracellular matrix
Source: Mol Psychiatry. 2023 Aug 29;28(11):4729–41. doi: 10.1038/s41380-023-02231-8 (PMC10914625; doi:10.1038/s41380-023-02231-8)
Supplement: Supplementary file 1 — Supplementary Information Key and Supplementary Figures [file 41380_2023_2231_MOESM1_ESM.docx]

-SUPPLEMENTARY INFORMATION FOR-

**Molecular Neurobiology of Loss: A Role for Basolateral Amygdala Extracellular Matrix**

Marissa A. Smail^1,2, #^, Brittany L. Smith^1^, Rammohan Shukla^3^, Khaled Alganem^3^, Hunter M. Eby^3^, Justin L. Bollinger^1^, Ria K. Parikh^1^, James B. Chambers^1^, James K. Reigle^4^, Rachel D. Moloney^5,6,7^, Nawshaba Nawreen^1^, Eric S. Wohleb^1^, Harry Pantazopoulos^8^, Robert E.

McCullumsmith^3,9,^^, James P. Herman^1,10,11,^^

**Table of Contents**

**Supplementary Methods**

**Supplementary Figures**

- **Supplementary Figure 1**: Bodyweights for Experiments 1-5
- **Supplementary Figure 2**: FOS Screen Results
- **Supplementary Figure 3**: Overview of Bioinformatics Analysis Pipeline
- **Supplementary Figure 4**: Full Results for Experiment 2 RNAseq
- **Supplementary Figure 5**: Full Results for Experiment 3 RNAseq
- **Supplementary Figure 6**: Full Results for Experiment 3 Proteomics and Kinomics
- **Supplementary Figure 7**: Additional IHC Results
- **Supplementary Figure 8**: Additional Behavior Results from Experiment 5
- **Supplementary Figure 9**: Additional Behavior Results from Experiment 6
- **Supplementary Figure 10**: Preliminary Female RNAseq Pathway Analysis

**Supplementary Tables** (*each as separate Excel file*):

- **Supplementary Table 1**: Overview of Experiments
- **Supplementary Table 2**: RNAseq Gene Expression Data
- **Supplementary Table 3**: Proteomics and Kinomics Data
- **Supplementary Table 4**: Pathway Analysis Results
- **Supplementary Table 5**: Perturbagen Analysis Results
- **Supplementary Table 6**: Cell Type Analysis Results
- **Supplementary Table 7**: Microglia and ECM Focused Gene Expression
- **Supplementary Table 8**: Detailed Statistical Results

**SUPPLEMENTARY METHODS**

**Subjects**

Eight-week-old adult male Sprague-Dawley rats were used for all experiments. Rats were obtained from Envigo (Indianapolis IN USA) at 8 weeks of age and given 1 week to acclimate to the vivarium prior to experimental manipulations. The vivarium was temperature and humidity controlled, and chow and water were provided ad libitum. There was a 12-hour light cycle (lights on at 8:00 AM, lights off at 8:00 PM). All procedures were conducted in compliance with the National Institutes of Health Guidelines for the Care and Use of Animals and approved by the University of Cincinnati Institutional Animal Care and Use Committee.

**Enrichment Removal Protocol**

Rats were randomly assigned (i.e., randomized into weight-matched groups) to 3 housing conditions: standard housing (SH), environmental enrichment (EE), and enrichment removal (ER). SH consisted of either single-housing (Exp 1-2) or pair-housing (Exp 3-5) (Supplementary Table 1). Prior experiments suggest that single and pair-housed male controls are highly similar, and that social isolation alone was insufficient to generate ER phenotypes[1]. EE rats remained in enrichment housing throughout the entire protocol. Enrichment housing consisted of 10 rats housed in a 1m^3^ multi-level wire mesh cage with 5-6 toys and shelters that rotated weekly (Figure 1A). Ten rats per cage was selected based on power analyses of prior experiments using this model[1, 2]. N was increased to 12 per group for surgery cohorts to account for hits and misses. Toys included plastic huts, tunnels, balls, chains, shovels, and buckets. While rats obtained physical enrichment from climbing and play, enrichment housing did not contain running wheels. As in previous studies[1, 2], EE rats were kept in enrichment housing during their active cycle (8 PM to 8 AM) and moved to single housing during the day (8 AM to 8 PM). ER rats were maintained in the same conditions as EE rats for 4 weeks. ER rats were then removed from EE and placed into 24-hour single housing for the remainder of the experiment, with no further enrichment. For molecular experiments, rats were kept naïve to behavioral testing and maintained on ER for 2 weeks prior to sacrifice (Figure 1A), ensuring that ER phenotypes have sufficient time to incubate[1]. These studies primarily focused on ER effects with SH serving as a procedural control group and EE serving as the canonical experiential control group.

**Experimental Design**

Six experiments were conducted for the present analyses (Supplementary Table 1). All experiments utilized the Enrichment Removal protocol, and procedural differences between studies will be noted where appropriate. All analyses were conducted by a blinded observer (i.e., analyses were conducted using animal numbers only and group assignments were not revealed until after analyses were completed). Note that in all six experiments ER rats gained more weight than EE and SH rats, demonstrating that the model was effective across all experiments (Supplementary Figure 1; Figure 6C). The experiments were conducted as follows:

***Experiment 1: Fos Immunohistochemistry Screen Following ER***

Fos expression profiling was first used to identify stress-responsive brain regions that showed differential activation in response to EE and ER (n=10/group). In this case, SH rats were kept in single housing (previous experiments did not document significant differences between single housing or pair housing on physiological or behavioral endpoints in male rats[1]), and EE rats were kept in enrichment housing 24 hours per day. These animals also experienced behavioral testing in the form of an open field test, social interaction test, and forced swim test (FST) starting 1 week after removal, conducted in 1-week intervals. Animals were sacrificed after FST to assess Fos protein activation to acute stress.

*Immunohistochemistry*

Rats were injected with an overdose of sodium pentobarbital 120 minutes after FST and transcardially perfused with 0.9% saline followed by 4% paraformaldehyde in 1xPBS. Brains were collected and placed in 4% paraformaldehyde overnight, after which they were placed in 30% sucrose solution (4 ºC). Brains were cut into 35µm serial sections using a sliding microtome and stored at -20ºC in cryoprotectant solution. Sections were removed from cryoprotectant, rinsed 5x5 min with 50mM KPS, treated with 1% hydrogen peroxide for 10 min, rinsed 5x5 min with KPBS, treated with 1% sodium borohydride (Fisher Scientific), rinsed 5x5 min with KPBS, and incubated for 1 hour in blocking solution (50mM KPBS, 0.1% BSA, 0.2% Triton X-100). Sections were transferred into wells with primary rabbit anti-cFOS antibody (Santa Cruz, no. sc-52, 1:5000) for incubation overnight. Sections were rinsed 5x5 min with KPBS, incubated for 1 hour in biotinylated anti-rabbit secondary antibody (Vector Laboratories Inc.), and rinsed 5x5 min in KPBS. Sections were incubated for 1 hour in avidin-biotin complex (Vector Laboratories Inc.) (1:800 in 50mMKPS + 0.1% BSA), rinsed 5x5 min in KPBS, and incubated for 10 min in 0.02% 3-diaminobenzidine (Sigma Aldrich) with 0.05% hydrogen peroxide. To terminate DAB incubation, sections were rinsed 4x5 min with KPBS. Finally, sections were mounted in 50mM phosphate buffer, dehydrated with an ethanol series, incubated in xylene for a minimum of 7 minutes and coverslipped with DPX (Sigma Aldrich).

*Image Analysis and Fos Quantification*

Images were obtained using a Zeiss Imager Z.1 (Carl Zeiss Microimaging) and 10x objective. The basolateral amygdala (BLA), medial amygdala (MEA), central amygdala (CEA), infralimbic prefrontal cortex (IL), prelimbic prefrontal cortex (PL), bed nucleus of the stria terminalis (BST), paraventricular nucleus (PVN), nucleus accumbens (NAc), and dentate gyrus (DG) were identified[3]. These regions have known roles in stress responsivity, making them likely candidates for dysregulation by chronic stress[4–6]. Bilateral images were collected at 3 levels for each region and quantified using Scion Image. A consistent threshold was used for all images to select and count immunolabeled cells in each ROI. The total number of Fos positive cells are expressed per arbitrary unit area for all regions except the MEA, which is expressed as number of Fos positive cells because area data was unavailable for this region.

*Statistics*

Normality and variance were assessed using GraphPad Prism 9. Group effects were analyzed by one-way ANOVA using Sigma Stat. All post hoc testing utilized Fisher’s Least Significant Difference (LSD). Based *a priori* on prior studies, outliers were determined by values that fall outside the mean ± 1.96 times the standard deviation[1, 2].

***Experiment 2: Case-Control RNAseq Analysis of the BLA***

Based upon the Fos screen, the BLA was selected for further analysis with RNAseq. This experiment was conducted on a new cohort of rats (n=10/group), and the ER protocol was run with active cycle enrichment. SH rats were kept in single housing. SH, EE, and ER rats were sacrificed by rapid decapitation 2 weeks after removal. Brains were collected, flash frozen in isopentane, and stored at -80⁰C.

*BLA Sample Collection*

Brains were blocked at the base of the cerebrum, mounted on the cryostat, and cut in 500 µm sections. Sections were mounted on slides and 1mm diameter micropunches were collected bilaterally from 3 sections containing BLA (Figure 1C; AP -1.5 to AP-3.0)[3]. Micropunches fell within the BLA and were consistently taken from the same section of the BLA. Micropunches were stored at -80⁰C. Six rats per group were used for further analysis. RNA was obtained using Qiagen RNAqueous-Micro isolation kits. RNA quantity and quality were assessed using a Nanodrop ND-1000 spectrophotometer.

*RNAseq Protocol*

RNAseq was performed by the Genomics, Epigenomics and Sequencing Core (GESC) at the University of Cincinnati. Briefly, ~400ng RNA per sample underwent polyA RNA isolation, library preparation, and cluster generation prior to sequencing on the Illumina HiSeq system. The sequencing conditions were single end 50 bases and 25 M reads per sample. Bioinformatics analyses of this data is detailed below.

***Experiment 3: Multi-Omics Validation and Expansion***

Building upon these BLA molecular profiles, a new cohort was generated to run parallel RNAseq, shotgun proteomics, and serine-threonine kinomics. This triple-omics approach not only served as a validation of Experiment 2 (i.e., reproducibility between cohorts), but also allowed us to examine ER-induced changes at multiple molecular levels (i.e., protein expression and activity). In this case, SH rats were kept in pair housing, as it serves as a more realistic control condition and enables future studies of sex differences[2]^,^[1]. The ER protocol was conducted as describe above (n=10/group), and rats were sacrificed by rapid decapitation 2 weeks after removal. Brains were collected, and micropunches of the BLA were collected as described in Experiment 2.

*Tissue Processing: Triple Prep*

We developed the “triple prep” protocol (Figure 1C) to simultaneously extract RNA and protein from the same tissue sample. Micropunches were first homogenized in 50 µl of MPER, Halt, and RNAse inhibitor (ThermoFisher) using a hand pestle. This homogenized sample was then split into 3 portions. Twenty-five µl of homogenate was used for RNA extraction, which was performed using Qiagen Mini Kit according to the manufacturer’s protocol. Amount and quality of RNA was determined using a Nanodrop. All 260/280 values were greater than 1.8. The remaining twenty-five µl of homogenate was split for proteomics and kinomics analysis. The proteomics samples were used directly, while the kinomics samples were subjected to 10 minutes of centrifugation at 14,000 rpm and 4ºC. Amount of protein was assessed via BCA assay. All 3 types of samples were stored at -80ºC.

Following extractions, pools were made for each sample type and experimental group (n=10 individuals per pool). The logistics of using 3 platforms prohibited us from running individual samples, so representative pooled samples were generated for each group for each analysis. RNA samples were submitted to the Cincinnati Children’s Hospital Medical Center DNA Sequencing Core for RNAseq, protein samples were submitted to the University of Cincinnati Proteomics Core for Liquid Chromatography Mass Spectrometry (LCMS) shotgun proteomics, and protein samples were submitted to the University of Toledo Kinome Core for serine threonine kinomics.

*RNAseq Protocol*

Quality of RNA samples was assessed using an AATI Fragment Analyzer. All samples had an RQN greater than 8. Samples underwent RNA polyA stranded library preparation. RNA sequencing was performed via HiSeq 2500 Rapid Sequencing. The sequencing conditions were paired end 75 bases and 20 M reads per sample.

*Proteomics Protocol*

LCMS shotgun proteomics was used to run data-dependent acquisition (DDA) shotgun proteomics on the pooled protein samples. Three technical replicates were run for each sample (SH, EE, ER); however, due to technical difficulties only one replicate per group yielded usable data that underwent subsequent analyses.

*Kinomics Protocol*

The pooled protein samples were run in triplicate on PamGene Serine Threonine Kinase (STK) Arrays, with samples being equally represented across chips. This microarray approach measures phosphorylation of 144 peptides, and bioinformatics is used to infer upstream serine threonine kinase activity.

**Bioinformatics Analyses of Omics Data from Experiments 2 and 3**

Experiments 2 and 3 both yielded vast amounts of data regarding the molecular signatures of the BLA following EE and ER. Our primary goal here was to identify targets and pathways that replicated across cohorts and omics platforms. As such, we used various bioinformatics tools to summarize the data into meaningful functional profiles that could be easily compared (Supplementary Figure 3).

***RNAseq Analysis***

Bioinformatic analyses of RNAseq data were conducted in the same manner for Experiments 2 and 3. Raw fastq files were obtained from respective cores. Reads were aligned using HISAT2 and normalized using DESeq2. Fold change values were calculated for all detected protein-coding genes with an average base mean above 1. These differential expression values were generated for 3 contrasts (EEvSH, ERvSH, ERvEE) and pushed through our comprehensive analysis pipeline (Full Transcriptome Pathway Analysis, Targeted Pathway Analysis, and Signature Analysis). It is important to note that the following analyses are based on fold change (FC), rather than statistical significance. Given that Experiment 3 was run on pooled samples, we were unable to perform statistics and relied on FC to assess differential expression. Experiment 2 was also analyzed in this way to maintain consistency. This technique also emphasizes the functional implications of observed changes, which aligns with the goals of this project to identify biological substrates of loss.

*Full Transcriptome Pathway Analysis*

The first analysis utilized Gene Set Enrichment Analysis[7] (GSEA; gsea-msigdb.org) to assess transcriptome-wide pathway enrichment. GSEA uses a rank-ordered (by FC) list of all detected transcripts and compares that order to established pathway gene sets. If genes in a pathway are mainly high on the FC list, that pathway is positively enriched. Negatively enriched pathways show a high concentration of genes at the bottom of the FC list. GSEA was run using the fgsea R package and pathway gene sets were obtained from BaderLab (download.baderlab.org/EM_Genesets/). Gene sets used in this analysis contained between 15 and 500 genes, and GSEA was run with 10,000 permutations. GSEA generates p values and enrichment scores (ES) to assess the significance and strength of enrichment. Pathways with p<0.05 were extracted and condensed into functional categories using a new tool we developed called PathwayHunter[8].

While GSEA is a great tool for revealing subtle transcriptome-wide changes, it yields thousands of pathways, making it difficult to ascertain functional meaning. PathwayHunter was designed to take those thousands of pathways and summarize them into enriched categories. These categories were generated from comparison of the semantic similarity of the 44,000 Gene Ontology pathway titles and descriptions, which sorted them into 500 functionally related clusters. PathwayHunter then uses hypergeometric overlap to determine which of these clusters are overrepresented in the significant pathways from the dataset. It assigns a -log10 p value to demonstrate the magnitude of this overrepresentation (with higher scores indicating stronger involvement), along with a 2-word label to describe the category. An additional step of manual curation based on *a priori* knowledge was used to more broadly group overrepresented categories into functional themes. These themes can then be examined with GSEA ESs to determine which types of pathways were altered in which direction across groups.

Additionally, leading-edge genes, which are genes that are driving the observed pathways, were collected from GSEA. Cell-type enrichment of top 100 upregulated and downregulated leading- edge genes was then conducted in Kaleidoscope, using the “Brain RNA-seq module,” to determine if certain cell types were preferentially impacted by ER.

*Targeted Pathway Analysis*

The second analysis utilized Enrichr[9] (maayanlab.cloud/Enrichr/) to assess pathway enrichment within the top 100 differentially expressed genes (DEGs). This more traditional pathway analysis is designed to detect pathways with the strongest enrichment. The top 100 upregulated DEGs (by FC) were extracted and entered into the Enrichr R package, where significantly (p<0.05) upregulated pathways were collected for GO Biological Process, GO Molecular Function, and GO Cellular Component. The same analysis was done for the top 100 downregulated genes, and GO terms were condensed by PathwayHunter as described above.

*Signature Analysis*

The third analysis utilized iLINCS[10] (ilincs.org/ilincs/) to identify compounds related to ER signatures. iLINCS utilizes the L1000, a set of 978 genes whose expression is known for thousands of cell line signatures upon exposure to various perturbagens. The L1000 was extracted from our RNAseq data and uploaded to iLINCS, which identified the top 20 discordant (signature reversing) and concordant (signature mimicking) perturbagens. Mechanism of action (MOA) was determined using the L1000 Fireworks Display to inform on pathways related to the perturbagens.

***Proteomics Analysis***

Differential expression values were calculated for peptides that were detected with 99% confidence in all groups and values were subjected to quartile normalization. Peptide sequences were converted into their corresponding proteins using PiNET (eh3.uc.edu/pinet/peptideToProtein). The top 100 upregulated and downregulated proteins (by FC) were input into Enrichr for targeted pathway analysis. Enrichr was chosen because the signatures were not large enough to benefit from GSEA. Significant pathways were condensed using PathwayHunter.

***Kinomics Analysis***

Phosphorylation levels within each group were obtained for the 144 peptides on the STK chip. Kinome Random Sampling Analyzer[11] (KRSA) was used to infer upstream kinase activity. This approach uses a permutation method to generate z-scores for each kinase. Kinases with a z-score > 1.5 were considered to be over-represented, and kinases with a z-score < -1.5 were considered to be under-represented. Additionally, targeted pathway analysis was run using Enrichr on the top differentially phosphorylated peptides (FC > 1.2) and condensed using PathwayHunter.

**Cross-Platform Summary**

The primary goal of these multi-level omics analyses was to identify commonly implicated targets and pathways (Supplementary Figure 3). Direct comparison between RNAseq (Experiments 2 and 3), proteomics, and kinomics was enabled by PathwayHunter, which revealed functional categories that were implicated across platforms, analyses, and cohorts. These categories were further defined and bolstered by the perturbagen, cell-type, and kinase results, which pointed to more specific targets within these categories. Commonly implicated results across these platforms and analyses formed the basis for the novel ER hypotheses investigated in subsequent cohorts.

***Experiment 4: Expanded Examination of BLA Microglia and Extracellular Matrix***

A new cohort of ER rats (n=10/group) was generated and perfused 2 weeks after removal. Brains were cut into 40µm serial sections using a sliding microtome and stored at -20ºC in cryoprotectant to allow for immunohistochemical (IHC) analysis of endpoints implicated by the multi-omics results.

*Antibodies*

Further investigation was conducted on microglia and the extracellular matrix (ECM). IBA1 (Wako, rabbit anti-IBA1, 019-19741, 1:1000), a common microglial marker, was used to determine microglia counts and morphology. CD68 (Abcam, mouse anti-CD68, ab31630, 1:500), a phagocytosis marker, was used to examine microglia phagocytic activity. Wisteria Floribunda Agglutinin (WFA) (Vector, biotinylated, B-1355, 1:1000) was used to label chondroitin sulfate proteoglycans (CSPGs), a primary component of the ECM. Given that a major element of ECM is perineuronal nets (PNNs), which primarily surround Parvalbumin (PV) interneurons, we also stained for PV (Sigma, mouse anti-PV, P3088, 1:2000). Follow-up studies utilized vGAT (Synaptic Systems, rabbit anti-vGAT, 131 002, 1:1000) and vGLUT (Synaptic Systems, rabbit anti-vGLUT, 135 303, 1:2000) to examine inhibitory and excitatory synaptic inputs onto PV cells, respectively. Secondary antibodies included Cy3 goat anti-rabbit (Thermo A10520, 1:500), Streptavidin AlexaFluor 488 (Thermo S11223, 1:500), and Cy5 goat anti-mouse (Thermo A10524, 1:500).

*Immunohistochemistry*

Four triple label IHCs (IBA1/WFA/PV, IBA1/WFA/CD68, WFA/PV/vGAT, and WFA/PV/vGLUT) were run using the following general protocol and above antibody concentrations. Sections were removed from cryoprotectant, rinsed 5x5 min with 1xPBS, blocked for 1 hour (0.1% BSA and 0.4% Triton in 1xPBS) at room temperature, then incubated in primary antibody in block overnight at 4ºC. Sections were then rinsed 5x5 min with 1xPBS and moved into secondary antibody in block for overnight incubation at 4ºC. Sections were rinsed at 5x5 min with 1xPBS, and mounted in 1xPBS with 5% gelatin. Slides were allowed to dry overnight before coverslipping with gelvatol (Sigma Aldrich, 10981). The only variation of the protocol was increasing the block for CD68 (blocking: 1% BSA, 5% NGS, and 0.4% Triton; antibody block: 1% BSA, 2.5% NGS, and 0.4% Triton).

*Image Analysis and Quantification*

Images were obtained using a Nikon C1 confocal microscope (Nikon Instruments Inc). For microglia and ECM analyses, z-stacks (0.4µm steps) of the IBA1/WFA/PV IHC were captured using the 20x objective. Images were taken bilaterally from 3 levels of the BLA (AP -2.0, -2.5, and -3.0), flattened into maximum intensity projections, and analyzed using ImageJ. Image settings were kept constant for all animals. All counts and measures were collected by a blinded observer. IBA1 endpoints included number, area, and soma size of microglia. WFA and PV endpoints included WFA area, WFA intensity, and the ratio of PV cells with and without PNNs.

For microglia morphology and phagocytosis analyses, z-stacks (0.2µm steps) of the IBA1/WFA/CD68 IHC were captured using the 40x objective with a 2x zoom and included 2 bilateral levels of the BLA (AP -2.0, -2.5). CD68 area and area per microglia were quantified using ImageJ. Microglia morphology was assessed using the “filament tracer” tool in Imaris (n=8 cells/animal). This analysis was run using the default settings in Imaris, with primary endpoints including process area (i.e., filament dendrite area), process volume (i.e., filament dendrite volume), number of branches (i.e., filament no. dendrite branch pts), and number of terminal points (i.e., filament no. dendrite terminal pts).

For synaptic input analyses, z-stacks (0.2µm steps) of the WFA/PV/vGAT and WFA/PV/vGLUT IHCs were captured using the 60x objective and included 2 bilateral levels of the BLA (AP -2.0, -2.5). vGAT and vGLUT puncta apposed to PV cells were counted manually by a blinded observer using ImageJ and the Hyperstacks plugin. Given that this analysis was designed to determine the impact of PNNs on PV inputs, equal numbers of PV cells with and without PNNs were counted (n=8 PV cells/animal, 4 with PNNs, 4 without PNNs). This method masked group effects in favor of PNN effects, so data was collapsed across all 3 groups. Puncta within 1 µm of the cell’s perimeter were considered apposed, and puncta were counted at 3 levels for each cell[12]. Counts are presented as puncta/µm of PV perimeter. Additional endpoints included vGAT and vGLUT puncta intensity, PV intensity, PV perimeter, and vGAT/vGLUT ratio.

*Statistics*

Normality and variance were assessed, and group effects were analyzed by one-way ANOVA using GraphPad Prism 9. Data that were not normal were log transformed to achieve normality prior to statistical testing. All post hoc testing utilized Tukey tests. Outliers were determined by values that fall outside the mean ± 1.96 times the standard deviation. Pearson correlations were used to explore relationships between microglia and ECM endpoints.

***Experiment 5: Expanding BLA-Related Behavioral Profiling Following ER***

In order to tie our molecular results to behavioral outcomes, we generated two new cohorts of ER rats to 1) expand our behavioral profile of ER to behaviors known to centrally involve the BLA and 2) examine activation of BLA PV cells following behavioral testing.

**Behavioral Testing and Analysis**

BLA-related behavioral testing began 2 weeks after removal (Figure 1A). In the first cohort, testing consisted of passive avoidance[13, 14], three chamber social threat[15], and acoustic startle[16, 17] (Figure 5A). In the second, testing consisted of cued fear conditioning[13, 14] (Figure 5D). Testing was conducted between 8:00-14:00. Rats were sacrificed 90 minutes after the final task.

*Passive Avoidance*

Passive avoidance was run using the Gemini system (San Diego Instruments) and consisted of 5 phases: habituation, training, and 3 days of testing. The passive avoidance chamber features dark and light sides divided by a door. The door was open for the habituation phase, allowing exploration for 2 minutes. On training day, animals started in the light side, crossed to the dark side, received one mild shock, and were removed from the apparatus (0.5 mA). On testing days, animals started in the light side, the door was opened, and latency to cross to the dark side was measured automatically by the software. Animals that did not cross were removed after 10 minutes. In a few cases, animals were excluded due to technical errors with the behavioral equipment.

*Three Chamber Social Threat*

Social threat was assessed using a modified three chamber social interaction task. In this case, interactors were retired Long Evans breeders, representing an aggressive threat. The test consisted of 5 phases: habituation, training, and 3 days of testing. On habituation day, experimental animals were placed in the center chamber and allowed to free to explore for 10 minutes. The two outer chambers contained empty carousels during this phase. On training day, a Long Evans was placed in one of the carousels with a checkered wall providing a contextual cue in the aggressor’s chamber. The experimental animal was restricted to that chamber for the first 5 minutes. The door was then removed, and the experimental animal could leave and move freely for 5 minutes. On testing days, the carousels were empty with the checkered wall context around the prior aggressor chamber, and the experimental animal was placed in the center chamber and allowed to freely to explore for 10 minutes. At all stages, experimental animals were tracked using Ethovision 12 (Noldus) to determine the time spent in each chamber.

*Acoustic Startle*

Acoustic startle was run using the Gemini system (San Diego Instruments). This one-day test was conducted in an insulated chamber where animals were lightly restrained in a tube that measures startle response to auditory tones. Variable tones (0, 95, 110, 120 dB) were presented at random intervals for 24 trials, with each tone presenting 6 times. Average and max startle responses (mV) were calculated for each tone.

*Fear Conditioning*

Cued fear conditioning was run using Ethovision 12 (Noldus) and consisted of 5 phases: acquisition, 3 days of extinction, and reinstatement. Acquisition consisted of 6 shock-tone pairings (shock intensity 0.4 mA), each extinction day consisted of 7 tone presentations without a shock, and reinstatement (which was run 3 days after the final extinction day) consisted of 5 shock-tone pairings. Percent freezing was scored automatically each day using Ethovision’s tracking. Freezing was determined for the total trial, as well as specifically during tones and intertrial intervals (ITIs).

*Statistics*

Normality and variance were assessed, and One-way ANOVAs and Two-way repeated measures ANOVAs (GraphPad Prism 9) were used to assess behavioral endpoints. Outliers were determined by values that fall outside the mean ± 1.96 times the standard deviation. For passive avoidance, normality could not be achieved due to the bimodal nature of the distribution and the fact that this cohort was underpowered. Kruskal-Wallis non-parametric testing was therefore used to analyze crossing latency on each day. For social threat, Group x Day ANOVAs were used to assess time in the “threatening” side. For acoustic startle, one-way ANOVAs were used to assess startle responses to each of the tones. For fear conditioning, Group x Trial ANOVAs were used to analyze freezing during tones and ITIs on each day, and a Group x Day ANOVA was used to analyze average freezing across phases. All post hoc testing utilized Tukey tests.

**Fos Activation in the BLA Following Fear Conditioning**

We analyzed BLA Fos activation 90 minutes following reinstatement. Brains were collected and stored as described in Experiment 4.

*Immunohistochemistry, Image Analysis, and Quantification*

A triple label for WFA/PV/Fos was run using the above WFA and PV markers and a new Fos antibody (Santa Cruz, rabbit anti-Fos, sc-253, 1:1000). Fluorescence IHC was run and analyzed using the same protocol described in Experiment 4. BLA cells were determined to be positive or negative for WFA, PV, and Fos, yielding counts for all combinations. One and two-way ANOVAs, followed by Tukey’s post hocs, were used where appropriate.

***Experiment 6: Testing the Necessity of BLA PNNs in Loss-like Behaviors***

In order to test the necessity of BLA PNNs in ER, we utilized Chondroitinase ABC (ChABC), a bacterial enzyme which digests PNNs, to deplete BLA PNNs during removal and test if we could block the development of loss-like behaviors.

*ChABC Pilot*

ChABC digests PNNs rapidly following injection and then PNNs gradually repopulate over the next few months[18, 19]. A pilot in which rats received unilateral BLA ChABC injections (Sigma, Chondroitinase ABC from Proteus vulgaris, C3667) was run to establish the degree and timing of PNN depletion in our hands. Two doses (50 U/ml and 200 U/ml ChABC in 1xPBS) and volumes (500 nl and 1000 nl) were tested (n=6/group), and injections were counterbalanced for side. Half of the rats were sacrificed 4-5 days after surgery to determine the initial degree of PNN depletion, and the other half were sacrificed 3 weeks after surgery to determine the degree of PNN repopulation at that time. Brains were collected and WFA/PV IHCs were run as described above. WFA area was measured bilaterally, enabling comparison between ChABC injected and uninjected sides using paired t-tests. Altogether, ChABC resulted in a ~90% depletion at 4-5 days and remained at a ~60% depletion 3 weeks later (Supplementary Figure 9A). A 200 U/ml dose and 500 nl volume were determined to be the optimum parameters for the experiment.

*Experimental Design*

This experiment utilized a 2x2 design, with removal (EE vs ER) and treatment (VEH vs ChABC) as factors (n=12/group) (Figure 6A). EE was chosen as the control group because it represents the more informative control for loss. At the time of removal, all rats received bilateral injections of either ChABC or VEH into the BLA (500 nl/side of 200U/ml ChABC or 1xPBS; coordinates AP -2.6, ML +/- 5.0, DV -8.0). All were single housed for a 2-day recovery period following surgery, with EE rats being placed back into the active-cycle enrichment paradigm afterwards. ER rats remained single housed for the remainder of the experiment. This study was run in 2 cohorts each with equal representation from each group. Behavioral testing began 9-12 days following surgery and was concluded by 20-23 days after surgery to remain within the established window of PNN depletion. Bodyweight was monitored throughout this period.

Behaviors consisted of passive avoidance, acoustic startle, and forced swim, with a 2-day rest period between each. Passive avoidance and acoustic startle were run and analyzed as described in Experiment 5. The forced swim test was run as a 2-day test, with a 15-minute swim on Day 1 and a 6-minute swim on Day 2 (swim tube diameter 20 cm, water 25 ± 2 °C). Rats were sacrificed 90-minutes after the Day 2 swim. Swimming, immobility, climbing, and diving behaviors were hand-scored by a blind observer for the first 6 minutes of each test day[20]. Rats were sacrificed and perfused 90-minutes after the Day 2 swim. Brains were collected, and IHC was used to verify injection sites and label WFA as described in Experiment 4. WFA area was quantified in the BLA to validate the effectiveness of ChABC PNN depletion in this cohort.

*Statistics*

Normality and variance were assessed, and One-way and Two-way ANOVAs, followed by Tukey’s post hocs, were used to analyze each endpoint, where appropriate. Passive avoidance testing was better powered in this cohort and normality could be achieved via log transformation prior to parametric testing. Outliers were determined by values that fall outside the mean ± 1.96 times the standard deviation.

**Supplementary Methods References**

1. Smith BL, Lyons CE, Correa FG, Benoit SC, Myers B, Solomon MB, et al. Behavioral and physiological consequences of enrichment loss in rats. Psychoneuroendocrinology. 2017;77:37–46.

2. Morano R, Hoskins O, Smith BL, Herman JP. Loss of Environmental Enrichment Elicits Behavioral and Physiological Dysregulation in Female Rats. Front Behav Neurosci. 2019;12:287.

3. Paxinos G, Charles Watson. The Rat Brain in Stereotaxic Coordinates Sixth Edition. 2007.

4. Ulrich-Lai YM, Herman JP. Neural regulation of endocrine and autonomic stress responses. Nat Rev Neurosci. 2009;10:397–409.

5. Nawreen N, Cotella EM, Morano R, Mahbod P, Dalal KS, Fitzgerald M, et al. Chemogenetic Inhibition of Infralimbic Prefrontal Cortex GABAergic Parvalbumin Interneurons Attenuates the Impact of Chronic Stress in Male Mice. ENeuro. 2020;7.

6. Wulsin AC, Kraus KL, Gaitonde KD, Suru V, Arafa SR, Packard BA, et al. The glucocorticoid receptor specific modulator CORT108297 reduces brain pathology following status epilepticus. Exp Neurol. 2021;341.

7. Broad Institute. Gene Set Enrichment Analysis. 2019. http://software.broadinstitute.org/gsea/index.jsp.

8. Wu X, Shukla R, Alganem K, Zhang X, Eby HM, Devine EA, et al. Transcriptional profile of pyramidal neurons in chronic schizophrenia reveals lamina-specific dysfunction of neuronal immunity. https://doi.org/10.1038/s41380-021-01205-y.

9. Ma’ayan Lab. Enrichr. 2019. https://amp.pharm.mssm.edu/Enrichr/.

10. NIH LINCS Program. iLINCS (integrative LINCS) genomics data portal. 2019. http://www.ilincs.org/ilincs/.

11. DePasquale EAK, Alganem K, Bentea E, Nawreen N, McGuire JL, Tomar T, et al. KRSA: An R package and R Shiny web application for an end-to-end upstream kinase analysis of kinome array data. PLoS One. 2021;16:e0260440.

12. Carceller H, Guirado R, Ripolles-Campos E, Teruel-Marti V, Nacher J. Perineuronal Nets Regulate the Inhibitory Perisomatic Input onto Parvalbumin Interneurons and c Activity in the Prefrontal Cortex. J Neurosci. 2020;40:5008–5018.

13. Sah P, Faber ESL, De Armentia ML, Power J. The amygdaloid complex: Anatomy and physiology. Physiol Rev. 2003;83:803–834.

14. Tovote P, Fadok JP, Lüthi A. Neuronal circuits for fear and anxiety. Nat Publ Gr. 2015;16:317.

15. Adolphs R. What does the amygdala contribute to social cognition? Ann N Y Acad Sci. 2010;1191:42–61.

16. Davis M, Walker DL, Lee Y. Roles of the amygdala and bed nucleus of the stria terminalis in fear and anxiety measured with the acoustic startle reflex. Possible relevance to PTSD. Ann N Y Acad Sci. 1997;821:305–331.

17. Janak PH, Tye KM. From circuits to behaviour in the amygdala. Nature. 2015;517:284–292.

18. Carulli D, Verhaagen J. An Extracellular Perspective on CNS Maturation: Perineuronal Nets and the Control of Plasticity. Int J Mol Sci 2021, Vol 22, Page 2434. 2021;22:2434.

19. Reichelt AC, Hare DJ, Bussey TJ, Saksida LM. Perineuronal Nets: Plasticity, Protection, and Therapeutic Potential. Trends Neurosci. 2019;42:458–470.

20. Slattery DA, Cryan JF. Using the rat forced swim test to assess antidepressant-like activity in rodents. Nat Protoc 2012 76. 2012;7:1009–1014.

**SUPPLEMENTARY FIGURES**

**
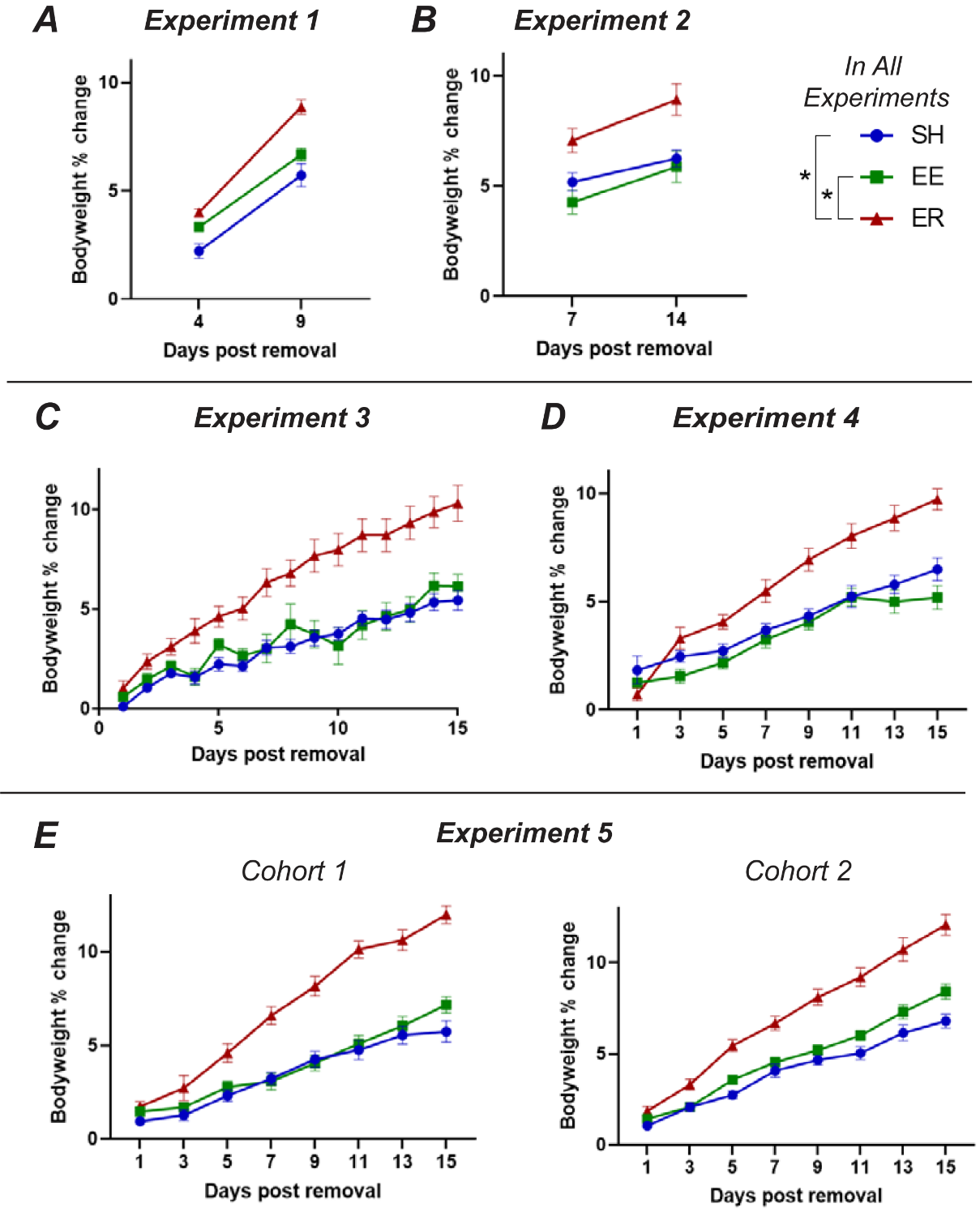
**

**Supplementary Figure 1: Bodyweights for Experiments 1-5.** ER consistently causes rats to gain weight (A-E) (n=10/group; Exp 5 Cohort 2 SH n=8, EE n=9, ER n=9). Data are presented as bodyweight percent change from weight at the time of enrichment removal. Note x-axis as days post removal, as visual differences here are due to differences in weight sampling times, not differences between experiments. In all experiments, ER rats gained more weight than EE and SH rats. * = p<0.05.

**
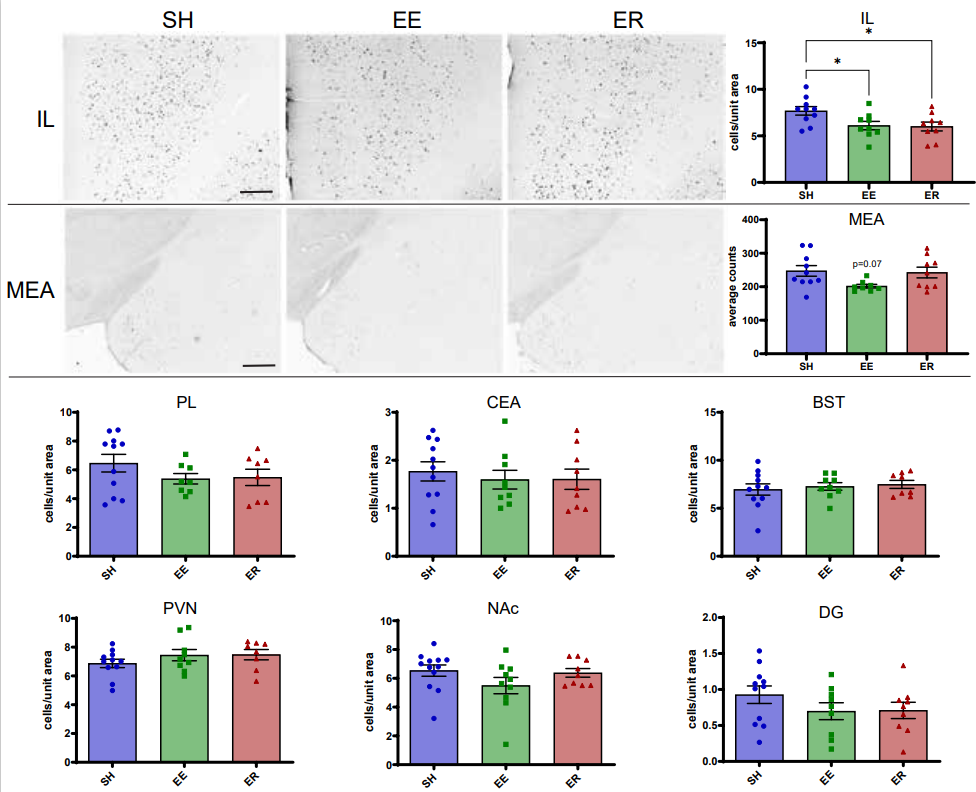
Supplementary Figure 2: FOS Screen Results.** SH, EE, and ER rats were sacrificed 120 minutes after the forced swim test. IHC was used to explore the FOS response in various stress-responsive brain regions. Representative images are shown for the regions that showed the most differential response between groups. * = p<0.05. IL = infralimbic prefrontal cortex (SH n=9, EE n=8, ER n=8). MEA = medial amygdala (SH n=10, EE n=8, ER n=9). PL = prelimbic prefrontal cortex (SH n=10, EE n=8, ER n=8). CEA = central amygdala (SH n=10, EE n=9, ER n=9). BST = bed nucleus of the stria terminalis (SH n=10, EE n=9, ER n=8). PVN = paraventricular nucleus (SH n=10, EE n=9, ER n=8). NAc = nucleus accumbens (SH n=10, EE n=9, ER n=9). DG = dentate gyrus (SH n=10, EE n=9, ER n=9).

**
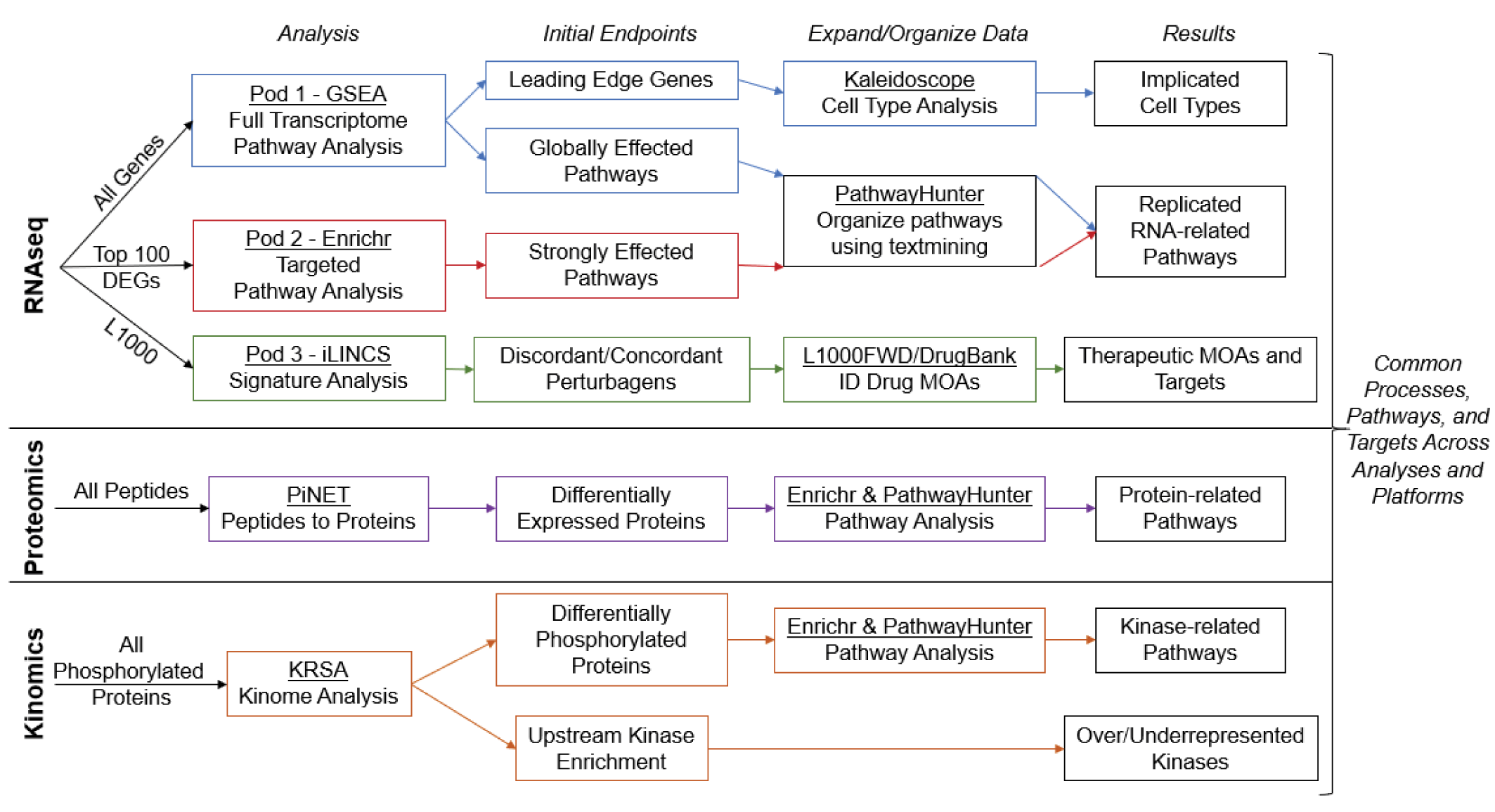
Supplementary Figure 3: Overview of Bioinformatics Analysis Pipeline.** Multi-Omics analyses were conducted as depicted from left to right, with data inputs, bioinformatics tools used, data condensing steps, and endpoints shown where appropriate. The ultimate goal of these analyses was to identify commonly implicated processes, pathways, and targets, with the notion that cross-analysis and cross-platform validation will yield higher-confidence results.

**
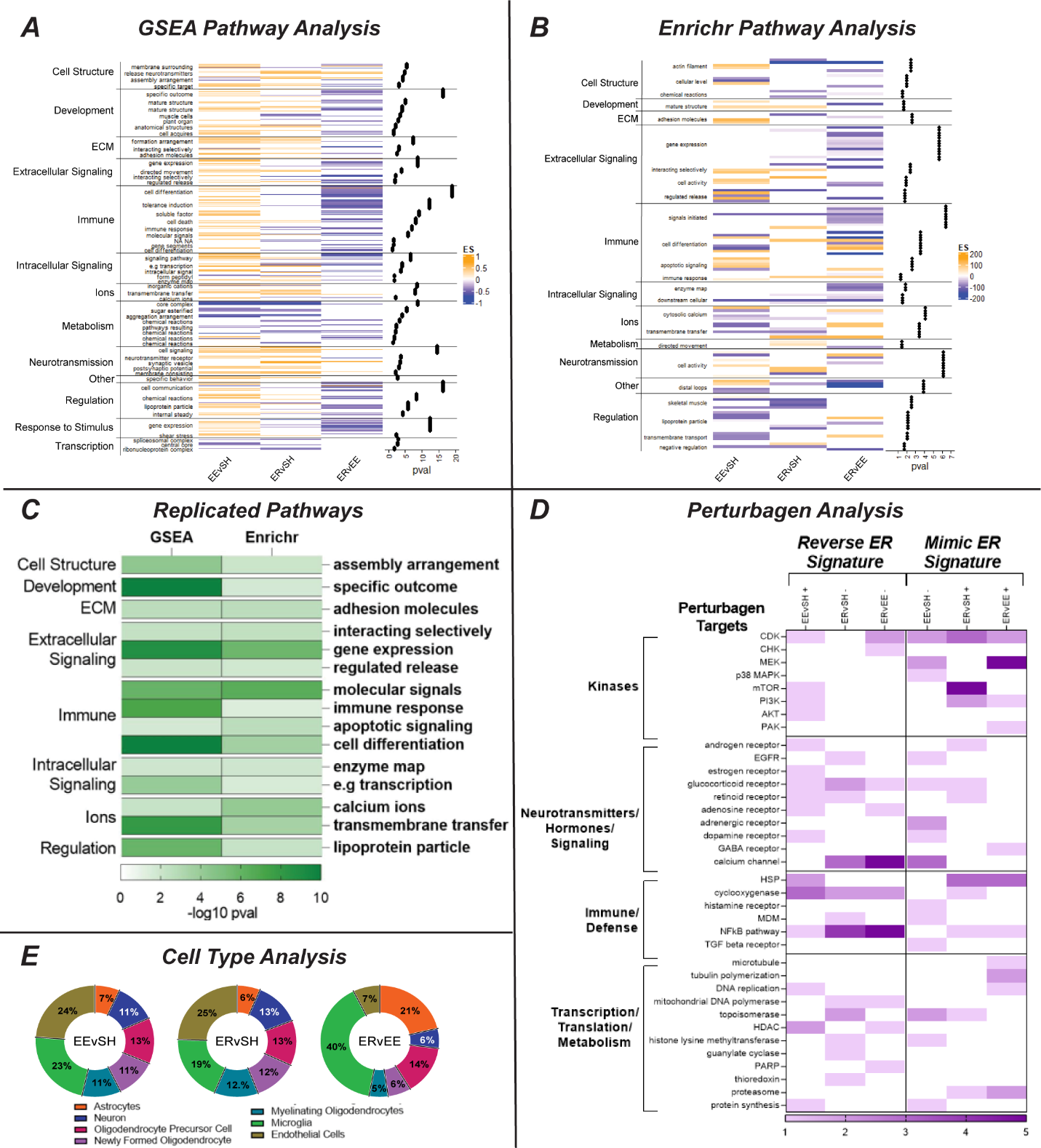
Supplementary Figure 4: Full Results for Experiment 2 RNAseq.** (A) Full transcriptome pathway analysis results. Significant pathways (p<0.05) from GSEA were condensed based on semantic similarity using Pathway Hunter (small left labels, and these categories were further condensed into functional themes based on *a priori* knowledge (leftmost labels). Enrichment scores for individual pathways are represented by the heatmap (with EEvSH on the left, ERvSH in the middle, and ERvEE on the right), and the degree of each category’s enrichment is represented by the p value dots on the right. The enrichment scores show how much (magnitude) and in what direction (yellow is up, blue is down) a theme was changed by the model, while the p values show the contribution of each category to these changes. (B) Targeted pathway analysis results. Analysis is the same as described in A but uses significant pathways (p<0.05) from Enrichr. (C) Themes that replicated between these two pathway analyses are shown in the replicated pathways heatmap, with darker green indicating more involvement. Right labels are Pathway Hunter categories, and left labels are their larger themes. (D) Signature analysis results. iLINCS perturbagen signatures that are concordant with EEvSH or discordant with ERvSH and ERvEE would theoretically reverse ER, while signatures that are discordant with EEvSH or concordant with ERvSH and ERvEE would theoretically mimic ER. MOAs (small left labels) for the top 20 perturbagens were organized into functional themes (far left label). Purple represents the number of perturbagens with that MOA in that contrast. (E) Cell type analysis results.

**
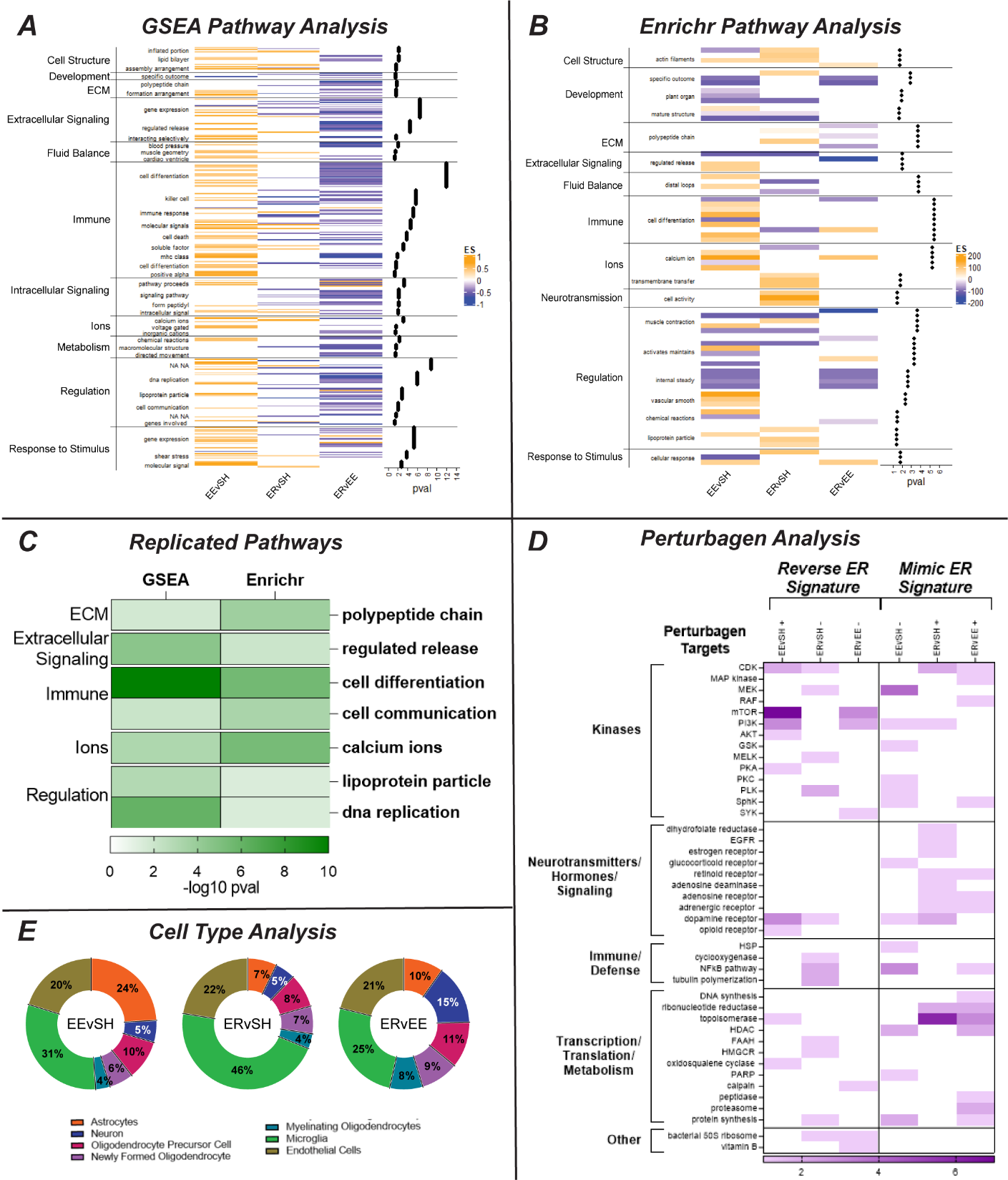
Supplementary Figure 5: Full Results for Experiment 3 RNAseq.** Data is presented as described in Supplementary Figure 4. (A) Full transcriptome pathway analysis results. (B) Targeted pathway analysis results. (C) Replicated pathways. (D) Signature analysis results. (E) Cell type analysis results.

**
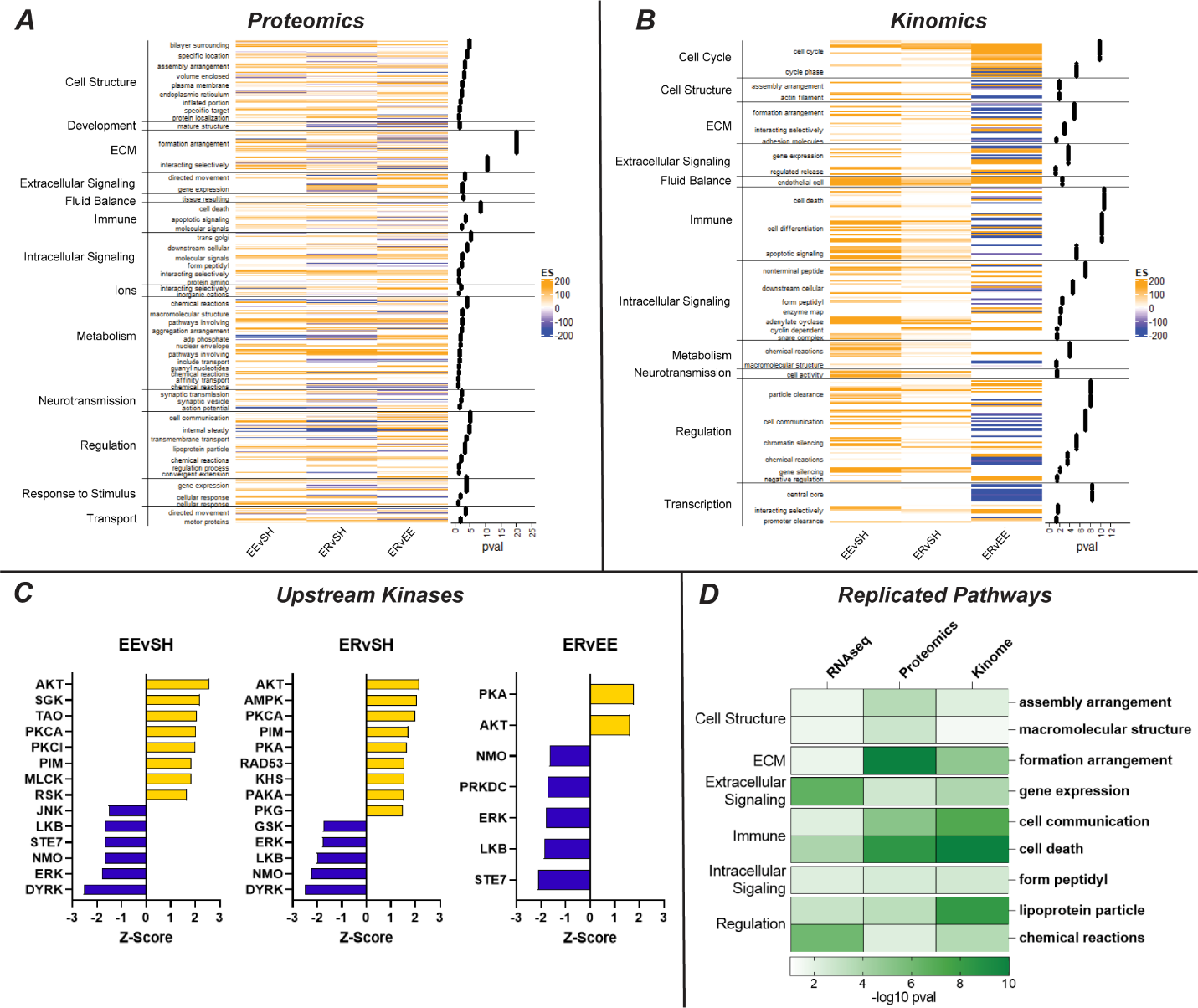
Supplementary Figure 6: Full Results for Experiment 3 Proteomics and Kinomics.**  (A) Proteomics pathway results. Pathways significantly enriched (p<0.05) in the top 100 upregulated and top 100 downregulated peptides. Data is presented as described in Supplementary Figure 4A. (B) Kinomics pathway results. Pathways significantly enriched (p<0.05) in the differentially phosphorylated (log2FC>0.3) peptides. Data is presented as described in Supplementary Figure 4A. (C) Upstream kinases identified by KRSA. Z-scores are presented for each kinase in each group, with higher z-scores indicating stronger over- (yellow) or under- (blue) representation. (D) Replicated pathways across Experiment 3 RNAseq, proteomics, and kinomics. Data is presented as described in Supplementary Figure 4C.

**
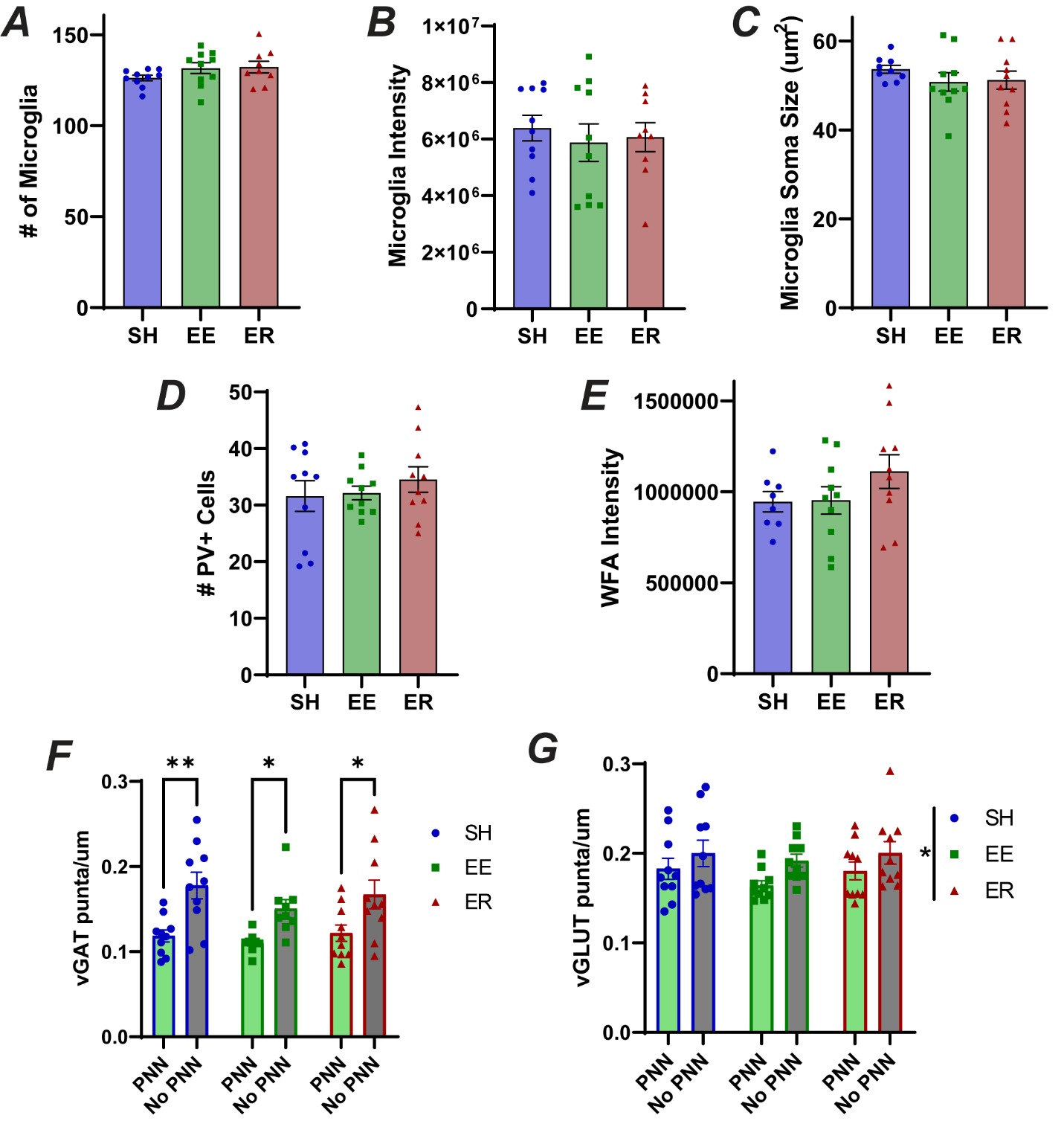
Supplementary Figure 7: Additional IHC Results.** Other BLA endpoints from Experiments 4 and 5. (A) Microglia counts (SH n=10, EE n=10, ER n=9). (B) Microglia intensity (n=10/group). (C) Microglia soma size (n=10/group). (D) Parvalbumin counts (n=10/group). (E) WFA intensity (n=10/group). No differences were observed in these measures. (F) vGAT puncta counts separated by group (n=10/group). (G) vGLUT puncta counts separated by group (n=10/group). Note that PNN phenotypes were observed in all conditions. * = p<0.05

**
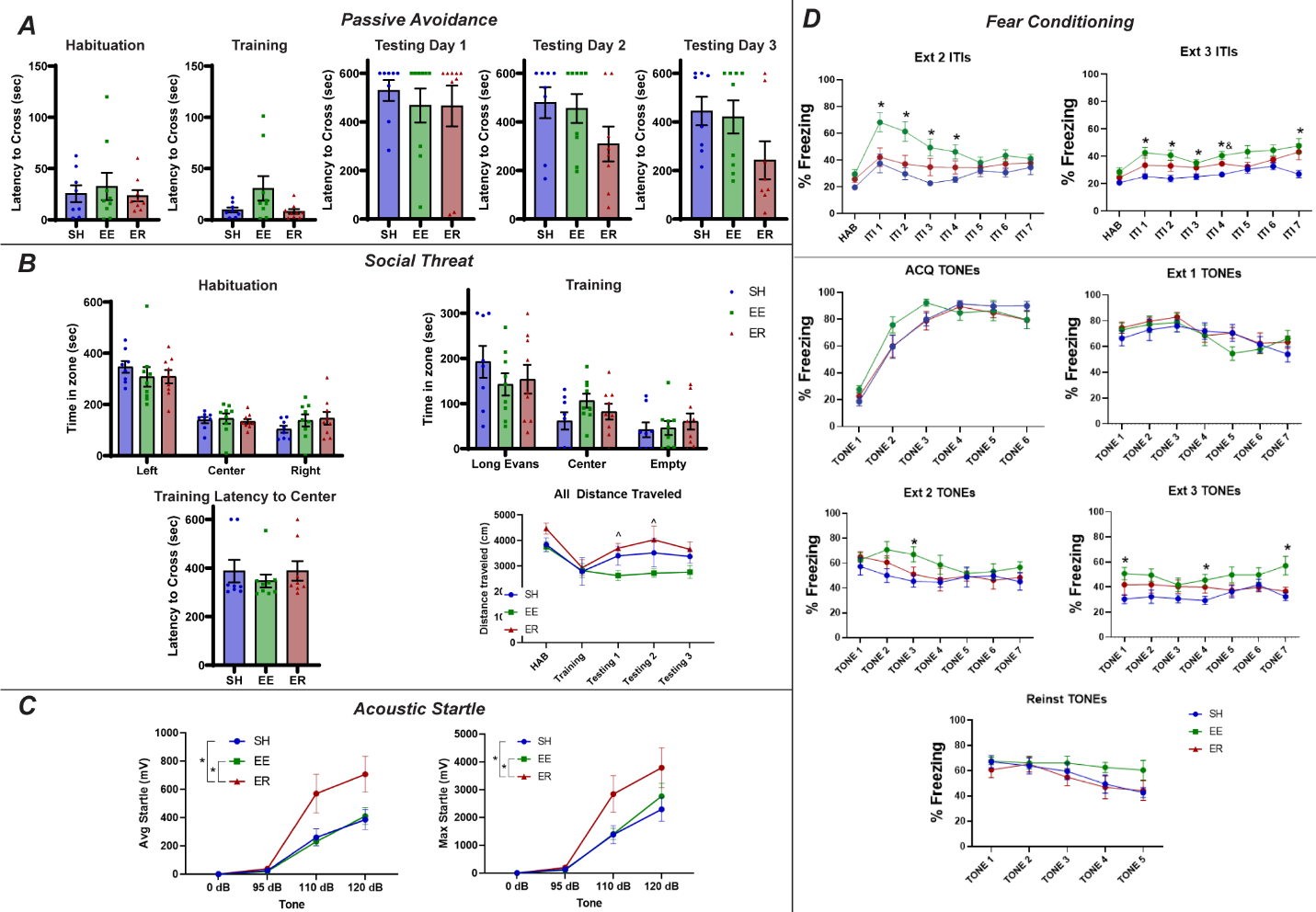
Supplementary Figure 8: Additional Behavior Results from Experiment 5.** (A) Passive avoidance latency to cross on habituation, training, and testing days (SH n=8, EE n=9, ER n=9). No significant differences were observed. (B) Additional social threat endpoints, including time in each zone on habituation and training days, time to leave the retired breeder long evans (LE) zone on training day, and overall locomotion (SH n=8, EE n=9, ER n=9). All groups showed a bias for the left compartment that persisted across days. The presence of the LE caused EE animals to spend less time in this zone. No differences were observed in time to leave the LE zone. ER rats did travel a greater distance than EE rats on all testing days, possibly pointing to increased escape behavior. (C) Average and maximum startle intensity across tones (SH n=8, EE n=9, ER n=9). ER rats responded more to the higher tones for both endpoints. (D) Fear Conditioning extinction day 2 and 3 freezing during it is (n=10/group). ER and SH rats showed enhanced extinction compared to EE rats. Freezing during fear conditioning tones each day. Patterns are similar to those seen during ITIs, but of a smaller magnitude. * = p<0.05

**
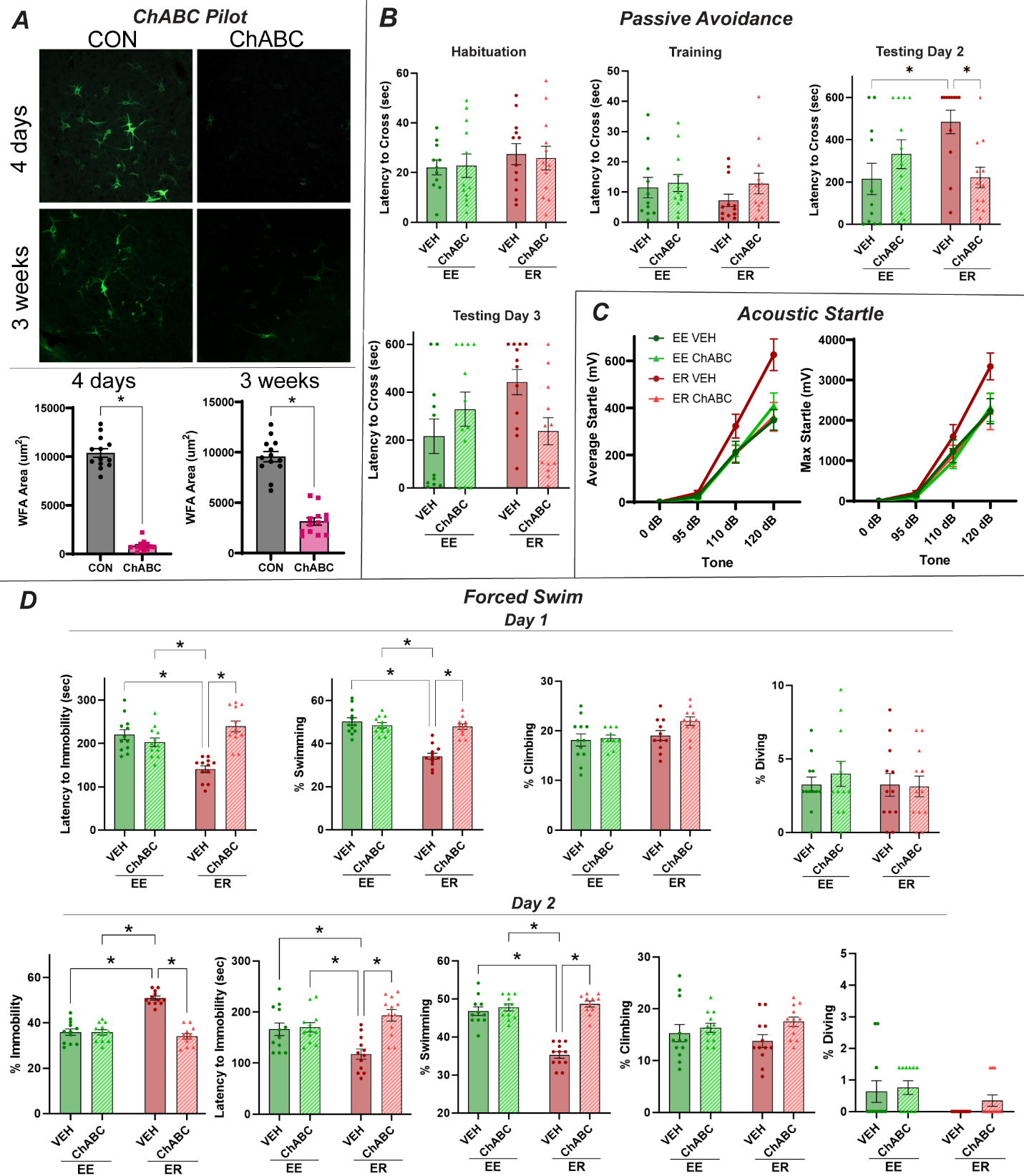
Supplementary Figure 9: Additional Results from Experiment 6.** (A) ChABC pilot. BLA WFA area was reduced ~90% 4 days following ChABC injection and remained ~60% depleted 3 weeks later (n=12/group). (B) Passive avoidance latency to cross on habituation, training, and testing days 2 and 3 (n=12/group). Other testing days exhibited the same pattern observed on testing day 1 (Figure 6D). ER VEH rats showing an increased latency to cross, with ChABC blocking this effect. (C) Average and maximum startle intensity across tones (n=12/group). ER rats responded more to the highest tone for both endpoints. (D) Additional forced swim test results (n=12/group). ER VEH rats showed an increased latency to immobility and decreased swimming. ChABC blocked both effects in ER rats. No differences were observed for climbing and diving behaviors. The same patterns were observed on Day 2, with a slight shift toward increased mobility observed across all groups. * = p<0.05

**
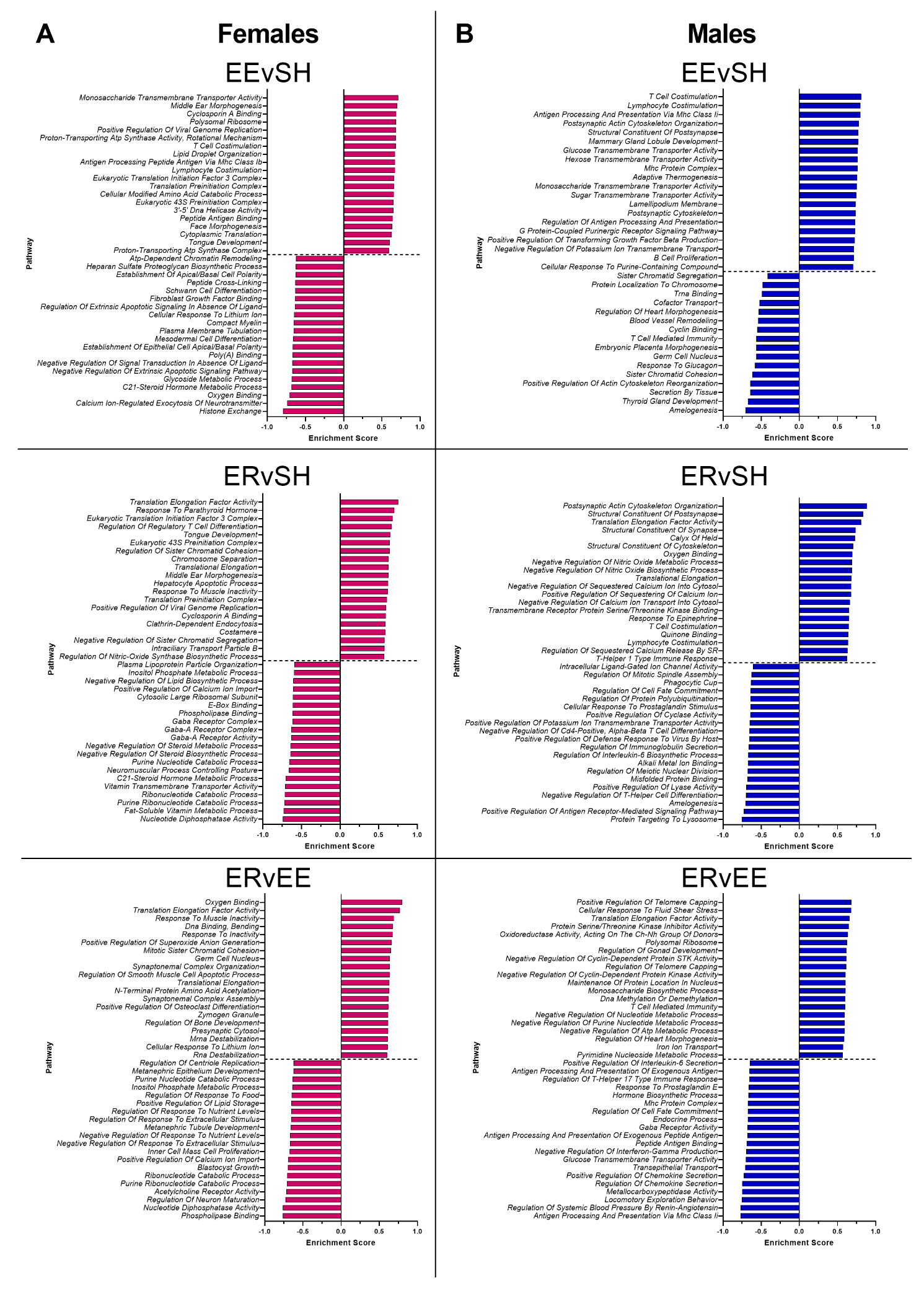
Supplementary Figure 10: Preliminary Female BLA RNAseq Pathway Analysis.** (A) A female cohort was run parallel to the Experiment 3 male cohort, and RNAseq was run on pooled samples from the BLA. GSEA full transcriptome pathway analysis was run as described above, and the top 20 significantly enriched up- and down-regulated pathways are presented here, along with respective enrichment scores (ES). (B) Male top 20 GSEA pathways for comparison. Note the differences between female and male pathways. Females demonstrated greater involvement of metabolic, transcriptional, and developmental processes, whereas males demonstrated greater involvement of immune and extracellular signaling***.***

**SUPPLEMENTARY TABLES** *(each as separate Excel file)*

**Supplementary Table 1: Overview of Experiments.** This study consisted of 6 experiments. The goals and approaches of each experiment are detailed here.

**Supplementary Table 2**: **RNAseq Gene Expression Data.**

*Tab 1:*  Experiment 2 *Differential Gene Expression*. Experiment 2 RNAseq differential expression values for EEvSH, ERvSH, and ERvEE genes. Gene names are provided in column A, and differential expression values are presented in log2FC.

*Tab 2:*  Experiment 3 *Differential Gene Expression*. Experiment 3 RNAseq differential expression values for EEvSH, ERvSH, and ERvEE genes. Gene names are provided in column A, and differential expression values are presented in log2FC.

**Supplementary Table 3: Proteomics and Kinomics Data.**

*Tab 1: Proteomics Differential Expression.*  Differential expression of peptides detected via LCMS, presented in log2FC.

*Tab 2: Kinomics Differential Phosphorylation.*  Differential phosphorylation of STK peptides, presented in FC.

*Tab 3: Kinomics KRSA.* Upstream kinase analysis. Z-score signifies how over- (positive) or under- (negative) represented a particular kinase’s activity is in that contrast. Kinomics was run in triplicate on 3 separate STK chips, so z-scores are provided for each chip and as an overall average.

*Tab 4: Kinomics KRSA Summary*. Summary of average z-scores from Tab 3 for kinases considered to be strongly represented (>2.0), represented (1.75-2), or weakly represented (1.5-1.75). Kinases with z-scores <1.5 are not considered to be differentially active in the sample. Corresponds to Supplementary Figure 6C.

**Supplementary Table 4: Pathway Analysis Results**

*Tab 1: Experiment 2 GSEA Pathways*. Detailed pathways for Experiment 2’s RNAseq Full Transcriptome Pathway Analysis. Corresponds to Figure 2A and Supplementary Figure4A. IDs and titles of Individual pathways are provided in columns D and E, respectively. Enrichment Scores for that pathway in each contrast are provided in columns F-H, with yellow signifying positive scores, blue signifying negative scores, and while signifying that that pathway was not significantly enriched in that contrast. Pathways are organized into clusters based on semantic similarity by PathwayHunter. PathwayHunter cluster names, numbers, and p values (how enriched that cluster is overall) are provided in columns B, C, and I. The larger themes that these categories were organized into based on *a priori* knowledge are provided in column A. This file can be used to look up which pathways make up the categories and themes presented in the main body of the manuscript.

*Tab 2: Experiment 2 Enrichr Pathways.* Detailed pathways for Experiment 2’s RNAseq Targeted Pathway Analysis. Corresponds to Supplementary Figure 4B. See Tab 1 legend for key. Individual pathway enrichments are presented as Enrichr Combined Scores, rather than GSEA Enrichment Scores.

*Tab 3: Experiment 2 Replicated Pathways.* Themes of pathways observed in both GSEA and Enrichr results. Numbers correspond to -log10pvalue enrichment of PathwayHunter categories (on right). Overarching themes provided on left. Corresponds to Supplementary Figure 4C.

*Tab 4*: *Experiment 3 GSEA Pathways.* Detailed pathways for Experiment 3’s RNAseq Full Transcriptome Pathway Analysis. Corresponds to Supplementary Figure 5A. See Tab 1 legend for key.

*Tab 5: Experiment 3 Enrichr Pathways.* Detailed pathways for Experiment 2’s RNAseq Targeted Pathway Analysis. Corresponds to Supplementary Figure 5B. See Tab 2 legend for key.

*Tab 6*: Experiment 3 Replicated Pathways. Themes of pathways observed in both GSEA and Enrichr results. See Tab 3 legend for key. Corresponds to Supplementary Figure 5C.

*Tab 7: Experiment 3 Proteomics Pathways.* Detailed pathways for Experiment 2’s Proteomics Pathway Analysis. Corresponds to Supplementary Figure 6A. See Tab 2 legend for key.

*Tab 8: Experiment 3 Kinomics Pathways.* Detailed pathways for Experiment 2’s Kinomics Pathway Analysis. Corresponds to Supplementary Figure 6B. See Tab 2 legend for key.

*Tab 9: Experiment 3 All Replicated Pathways.* Themes of pathways observed in RNAseq, proteomics, and kinomics results. See Tab 3 legend for key. Corresponds to Supplementary Figure 6D.

*Tab 10: Overall Replicated Pathways.* Themes of pathways observed in Experiment 2 RNAseq results and Experiment 3 RNAseq, proteomics, and kinomics results. Corresponds to Figure 2E. See Tab 3 legend for key.

**Supplementary Table 5: Perturbagen Analysis Results**

*Tab 1: Experiment 2 Perturbagens*. Top 20 concordant (+) and discordant (-) perturbagens for EEvSH, ERvSH, and ERvEE. ID, name, and mechanism of action are given for each perturbagen, along with the p value and z-score representing the significance and strength of similarity between that perturbagen’s L1000 signature and the experimental L1000 signature.

*Tab 2: Experiment 2 Summary.* Summary of MOAs expected to reverse or mimic ER signatures. Numbers correspond to the number of perturbagens that had that MOA for each contrast, calculated from the individual perturbagens in Tab 1. Corresponds to Supplementary Figure 4D.

*Tab 3: Experiment 3 Perturbagens*. Top 20 concordant (+) and discordant (-) perturbagens for EEvSH, ERvSH, and ERvEE. ID, name, and mechanism of action are given for each perturbagen, along with the p value and z-score representing the significance and strength of similarity between that perturbagen’s L1000 signature and the experimental L1000 signature.

*Tab 4: Experiment 3 Summary.* Summary of MOAs expected to reverse or mimic ER signatures. Numbers correspond to the number of perturbagens that had that MOA for each contrast, calculated from the individual perturbagens in Tab 3. Corresponds to Supplementary Figure 5D.

**Supplementary Table 6: Cell Type Analysis Results**

*Tab 1: Experiment 2 Leading Edge Genes.* Top 100 upregulated and top 100 downregulated leading-edge genes from GSEA are provided for EEvSH, ERvSH, and ERvEE in columns B, L, and V, respectively. Expected expression of these genes in different cell types, named in row 2, are given next to each gene.

*Tab 2: Experiment 2 Average Cell Types.* Average leading-edge gene expression in different cell types, calculated from the individual gene values in Tab 1. Corresponds to Figure 3D and Supplementary Figure 4E.

*Tab 3: Experiment 3 Leading Edge Genes.* Top 100 upregulated and top 100 downregulated leading-edge genes from GSEA are provided for EEvSH, ERvSH, and ERvEE in columns B, L, and V, respectively. Expected expression of these genes in different cell types, named in row 2, are given next to each gene.

*Tab 4: Experiment 3 Average Cell Types.* Average leading-edge gene expression in different cell types calculated from the individual gene values in Tab 3. Corresponds to Supplementary Figure 5E.

**Supplementary Table 7: Microglia and ECM Focused Gene Expression.** Differential gene expression related to microglia and ECM. Corresponds to Figure 2F.

**Supplementary Table 8: Detailed Statistical Results.**

*Tab 1: Figure 1*. Statistical results for Figure 1B.

*Tab 2: Figure 3*. Statistical results for Figure 3A-C.

*Tab 3: Figure 4*. Statistical results for Figure 4B-D, F, G, I-K

*Tab 4: Figure 5*. Statistical results for Figure 5B-C, E, G-I.

*Tab 5: Figure 6*. Statistical results for Figure 6B-F.

*Tab 6: Supplementary Figure 1*. Statistical results for Supplementary Figure 1A-E.

*Tab 7: Supplementary Figure 2*. Statistical results for Supplementary Figure 2.

*Tab 8: Supplementary Figure 7*. Statistical results for Supplementary Figure 7A-G.

*Tab 9: Supplementary Figure 8*. Statistical results for Supplementary Figure 8A-D.

*Tab 10: Supplementary Figure 9*. Statistical results for Supplementary Figure 9A-D.

*Tab 11: Outliers.* Outliers removed from the present datasets with respective standard deviations from the mean.
